# Inhibitory mechanisms of decoy receptor 3 in cecal ligation and puncture-induced sepsis

**DOI:** 10.1128/mbio.00521-24

**Published:** 2024-05-03

**Authors:** Jingqian Su, Wenzhi Chen, Fen Zhou, Rui Li, Zhiyong Tong, Shun Wu, Zhen Ye, Yichao Zhang, Ben Lin, Xing Yu, Biyun Guan, Zhihua Feng, Kunsen Chen, Qi Chen, Long Chen

**Affiliations:** 1 Fujian Key Laboratory of Innate Immune Biology, Biomedical Research Center of South China, College of Life Science, Fujian Normal University, Fuzhou, China; 2 Institute of Edible Fungi, Fujian Academy of Agricultural Sciences, Fuzhou, Fujian, China; 3 Department of Neurosurgery & Neurocritical Care, Huashan Hospital, Fudan University, Shanghai, China; 4 Department of Gastroenterology, the First Affiliated Hospital of Fujian Medical University, Fuzhou, China; Cornell University, Ithaca, New York, USA; The University of Texas at Dallas, Richardson, Texas, USA

**Keywords:** gut microbiota, decoy receptor 3, sepsis, anti-inflammatory

## Abstract

Despite its high mortality, specific and effective drugs for sepsis are lacking. Decoy receptor 3 (DcR3) is a potential biomarker for the progression of inflammatory diseases. The recombinant human DcR3-Fc chimera protein (DcR3.Fc) suppresses inflammatory responses in mice with sepsis, which is critical for improving survival. The Fc region can exert detrimental effects on the patient, and endogenous peptides are highly conducive to clinical application. However, the mechanisms underlying the effects of DcR3 on sepsis are unknown. Herein, we aimed to demonstrate that DcR3 may be beneficial in treating sepsis and investigated its mechanism of action. Recombinant DcR3 was obtained *in vitro*. Postoperative DcR3 treatment was performed in mouse models of lipopolysaccharide- and cecal ligation and puncture (CLP)-induced sepsis, and their underlying molecular mechanisms were explored. DcR3 inhibited sustained excessive inflammation *in vitro*, increased the survival rate, reduced the proinflammatory cytokine levels, changed the circulating immune cell composition, regulated the gut microbiota, and induced short-chain fatty acid synthesis *in vivo*. Thus, DcR3 protects against CLP-induced sepsis by inhibiting the inflammatory response and apoptosis. Our study provides valuable insights into the molecular mechanisms associated with the protective effects of DcR3 against sepsis, paving the way for future clinical studies.

**IMPORTANCE:**

Sepsis affects millions of hospitalized patients worldwide each year, but there are no sepsis-specific drugs, which makes sepsis therapies urgently needed. Suppression of excessive inflammatory responses is important for improving the survival of patients with sepsis. Our results demonstrate that DcR3 ameliorates sepsis in mice by attenuating systematic inflammation and modulating gut microbiota, and unveil the molecular mechanism underlying its anti-inflammatory effect.

## INTRODUCTION

Sepsis is defined as an infection-induced, life-threatening disorder of organs and is caused by an imbalance between inflammation and immunosuppression (1). It is a major public health issue affecting approximately 18 million people worldwide annually and has a mortality rate of 28%–50%. Unfortunately, no specific drugs are available for the clinical treatment of sepsis (2, 3). While controlling excessive inflammation during infection has traditionally been a central treatment strategy, recent evidence indicates a more intricate interplay between pro-inflammatory and anti-inflammatory responses in sepsis. A previous study delved into the multifaceted nature of sepsis pathogenesis, which encompasses hyper-inflammation among other factors (4). Notably, recent literature, such as the work of Hotchkiss et al., underscores the mixed results observed in clinical trials of anti-inflammatory therapies. This emphasizes the need to explore a wider array of therapeutic approaches, including restorative immunotherapies that aim to rebalance the immune response rather than merely suppressing inflammation. While the reduction of proinflammatory cytokines has been a focal point, our comprehension of sepsis necessitates strategies that encompass the broader immunological landscape, including both pro-inflammatory and anti-inflammatory responses, to formulate more efficacious treatment modalities (5). Three main types of sepsis animal models have been established, including lipopolysaccharide (LPS) infusion, *Escherichia coli* infusion, and cecal ligation and puncture (CLP). Being the most realistic model of sepsis, CLP is widely used for research (6). In the CLP model, intestinal bacteria, fungi, and metabolites can enter the abdominal cavity, leading to abdominal infection and systemic septicemia (7). Organ inflammation is related to sepsis-associated intestinal microbiota imbalance; however, the mechanism through which changes in the intestinal microbiota affect sepsis pathogenesis remains unclear (8).

Decoy receptor 3 (DcR3), a 33 kDa glycosylated soluble factor belonging to the tumor necrosis factor (TNF) receptor superfamily, lacks a transmembrane domain (9). Acting as a decoy, DcR3 binds to and obstructs three pro-apoptotic ligands: Fas ligand (FasL/TNFSF6/CD95L), LIGHT (TNFSF14), and TNF-like molecule 1A (TL1A/TNFSF15). As a member of the TNF receptor superfamily, DcR3 reflects the nuanced role of TNF antagonists in sepsis. Despite promising pre-clinical results, clinical trials have yielded varied outcomes for TNF antagonists. The role of DcR3 extends to various inflammatory diseases, including sepsis, where it can serve as a biomarker for early diagnosis. Furthermore, the ability of DcR3 to inhibit apoptosis in immune cells, especially in the spleen and thymus, is crucial for the immune response during sepsis. Additionally, DcR3 has been found to suppress the inflammatory response, a critical facet of sepsis, However, the role of DcR3 in the pathogenesis or suppression of inflammatory responses remains to be elucidated (10). Most healthy individuals have miniscule serum DcR3 levels (11). As the cell damage and apoptosis rates increase, DcR3 is upregulated to maintain homeostasis (12). A moderate elevation of DcR3 can be observed in various malignancies, inflammatory conditions, and autoimmune disorders (13). Additionally, bacterial antigens, such as LPS and lipoteichoic acid, selectively induce DcR3 (14).

The biological effects of DcR3 have been explored in various animal models. Mice and rats lack the gene encoding DcR3 but maintain the downstream receptors. Transgenic mice overexpressing DcR3 and DcR3.Fc have been used to study their biological effects (11); however, some limitations remain. DcR3.Fc is a 57 kDa recombinant chimeric human protein, which is expressed at the C-terminus of the Fc region of human IgG1 based on the DNA sequence encoding human DcR3. DcR3.Fc suppresses inflammatory responses in mice suffering from sepsis (15). However, Fc regions can exert detrimental effect on patients. Through interaction with perivascular macrophages and microglia, Fc regions can cause inflammatory reactions (16, 17). Due to their short half-lives and low accumulation in tissues, endogenous peptides can reduce the risk of complications caused by their metabolites (18). Notably, the endogenous DcR3 concentration in a patient is not sufficient to exert an adequate anti-inflammatory effect (19, 20); thus, supplementation of DcR3 is required to exert potent anti-inflammatory effects.

In this study, we hypothesized that a sufficient concentration of DcR3 can effectively treat sepsis. Therefore, recombinant DcR3 was obtained *in vitro*. Furthermore, postoperative DcR3 treatments were performed in mouse models of sepsis induced by LPS and CLP, and their underlying molecular mechanisms were explored. This study provides essential evidence of the beneficial effect of DcR3 in sepsis, supporting its further clinical testing.

## RESULTS

### DcR3 modulated inflammatory cytokine expression in RAW264.7 cells

**Fig 1:**
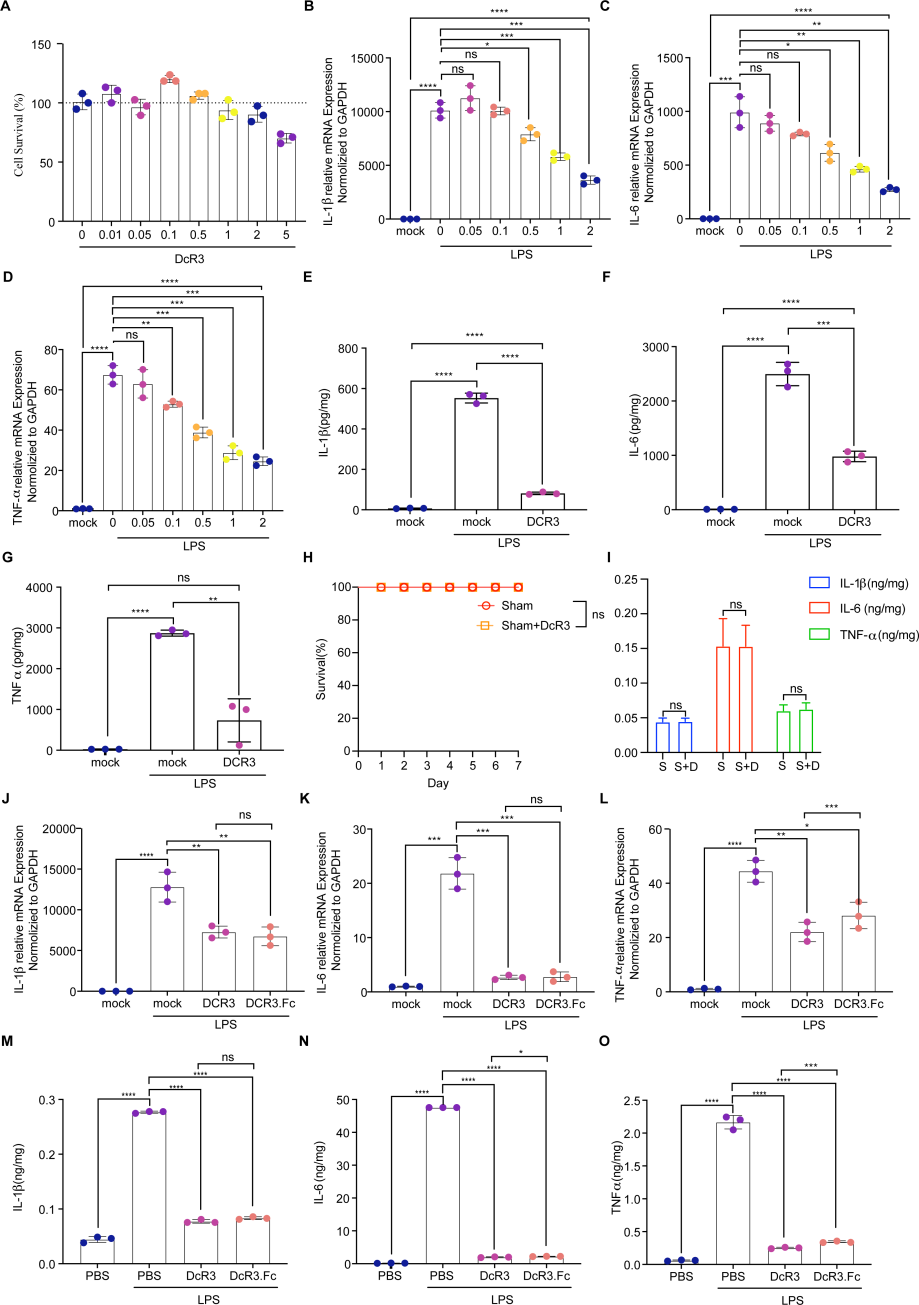
Effects of DcR3 on LPS-stimulated RAW264.7 cells and mice with LPS-induced sepsis. (**A**) Effect of DcR3 treatment (0–1.0 µg/mL) on cell viability. (**B–G**) RAW264.7 cells were pretreated with DcR3 treatment (0–2.0 µg/µL) and stimulated with LPS. (**B–D**) The mRNA levels of the cytokines *IL-6, TNF-α,* and *IL-1β* in RAW264.7 cells were measured using qPCR. (**E–G**) The levels of inflammatory cytokines (**E**) IL-1β, (**F**) IL-6, and (**G**) TNF-α determined using ELISA. (**H**) Effect of DcR3 on the survival of the Sham group (*n* = 10). (**I**) Effect of DcR3 on the expression levels of inflammatory factors in the serum of the Sham (S) group and Sham + DcR3 (S + D) group (*n* = 10). (**J–L**) Changes in mRNA levels of cytokines *IL-6, TNF-α,* and *IL-1β* in RAW264.7 cells following DcR3 treatment alone or co-treatment with LPS using qPCR. (**M–O**) Changes in the LPS-induced septic mice serum levels of inflammatory cytokines (**M**) IL-1β, (**N**) IL-6, and (**O**) TNF-α determined using ELISA at 12 h after DcR3 and DcR3.Fc treatment. ANOVA and Tukey’s post hoc test were used to analyze the data. (∗) *P* < 0.05, (∗∗) *P* < 0.01, (∗∗∗) *P* < 0.001, and (∗∗∗∗) *P* < 0.0001. An independent experiment was conducted thrice to produce the results.

The purified DcR3 (33 kDa) was validated using SDS-PAGE (Fig. S1 and S2). Results showed that the cytotoxicity of DcR3 (0–5.0 µg/µL) on RAW264.7 cells was negligible (Fig. 1A). Furthermore, DcR3 (1.0 µg/µL) significantly inhibited the upregulation of IL-1β, IL-6, and TNF-α induced by LPS in RAW264.7 cells (*P* < 0.01; Fig. 1B through G).

### DcR3 treatment in mice with LPS-induced sepsis

Initially, we administered DcR3 (1.0 mg/kg/d) to the Sham group to assess its effect on mouse survival and inflammatory marker expression (IL-1β, IL-6, and TNF-α). The findings revealed no significant impact of DcR3 on these parameters in the Sham group (*P* > 0.05, Fig. 1H and I). The therapeutic effect of DcR3 (1.0 mg/kg/d) and DcR3.Fc (1.0 mg/kg/d) was evaluated using the tissues from LPS-induced sepsis mouse models. Proinflammatory factors were significantly reduced by DcR3 and DcR3.Fc treatments (*P* < 0.0001, Fig. 1J through O).

### Therapeutic effect of DcR3 on mice with CLP-induced sepsis

**Fig 2:**
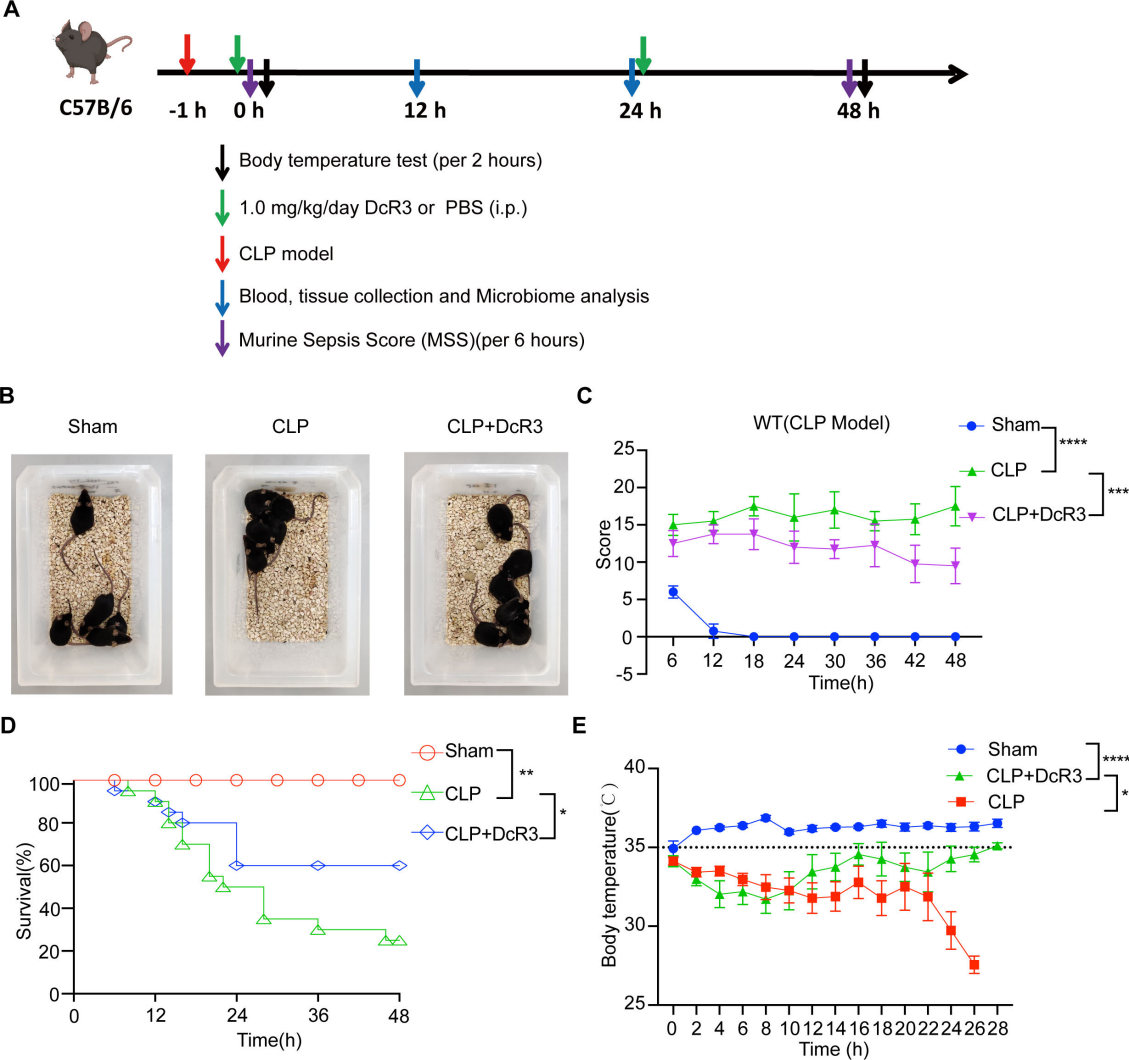
DcR3 displayed a significant therapeutic effect in mice with CLP-induced sepsis. (**A**) Experimental procedure timeline for generating CLP-induced sepsis mouse model. (**B**) Typical behavioral changes in septic mice after 12 h of DcR3 treatment. (**C**) The murine sepsis score (MSS) in septic mice (*n* = 6). (**D**) Effect of DcR3 on the survival of septic mice (*n* = 30). (**E**) Effect of DcR3 treatment on body temperature in septic mice. Data were statistically analyzed using the Mantel-Cox test (**B**), and two-way ANOVA followed by Bonferroni’s post hoc test (**C–E**). (∗) *P* < 0.05, (∗∗) *P* < 0.01, (∗∗∗) *P* < 0.001, and (∗∗∗∗) *P* < 0.0001. Sample sizes are indicated in brackets.

Subsequently, a CLP-induced sepsis mouse model was employed to determine DcR3’s therapeutic potential (Fig. 2A). Compared to the untreated CLP group, the DcR3-treated group showed reduced agglomeration and improved physical condition, activity, and appetite (Fig. 2B). DcR3 treatment significantly reduced the murine sepsis scores (MSSs) induced by CLP (*P* < 0.001, Fig. 2C). A higher survival rate was observed in the DcR3-treated group than in the CLP group (60% vs 25%, *P* < 0.05, Fig. 2D). After 26 h of sepsis, the body temperature of mice in the DcR3-treated group rapidly returned to normal (*P* < 0.05, Fig. 2E). These findings indicate that DcR3 might play an important therapeutic role in mice with CLP-induced sepsis.

### DcR3 reduced inflammatory cytokine levels in mice with CLP-induced sepsis

**Fig 3:**
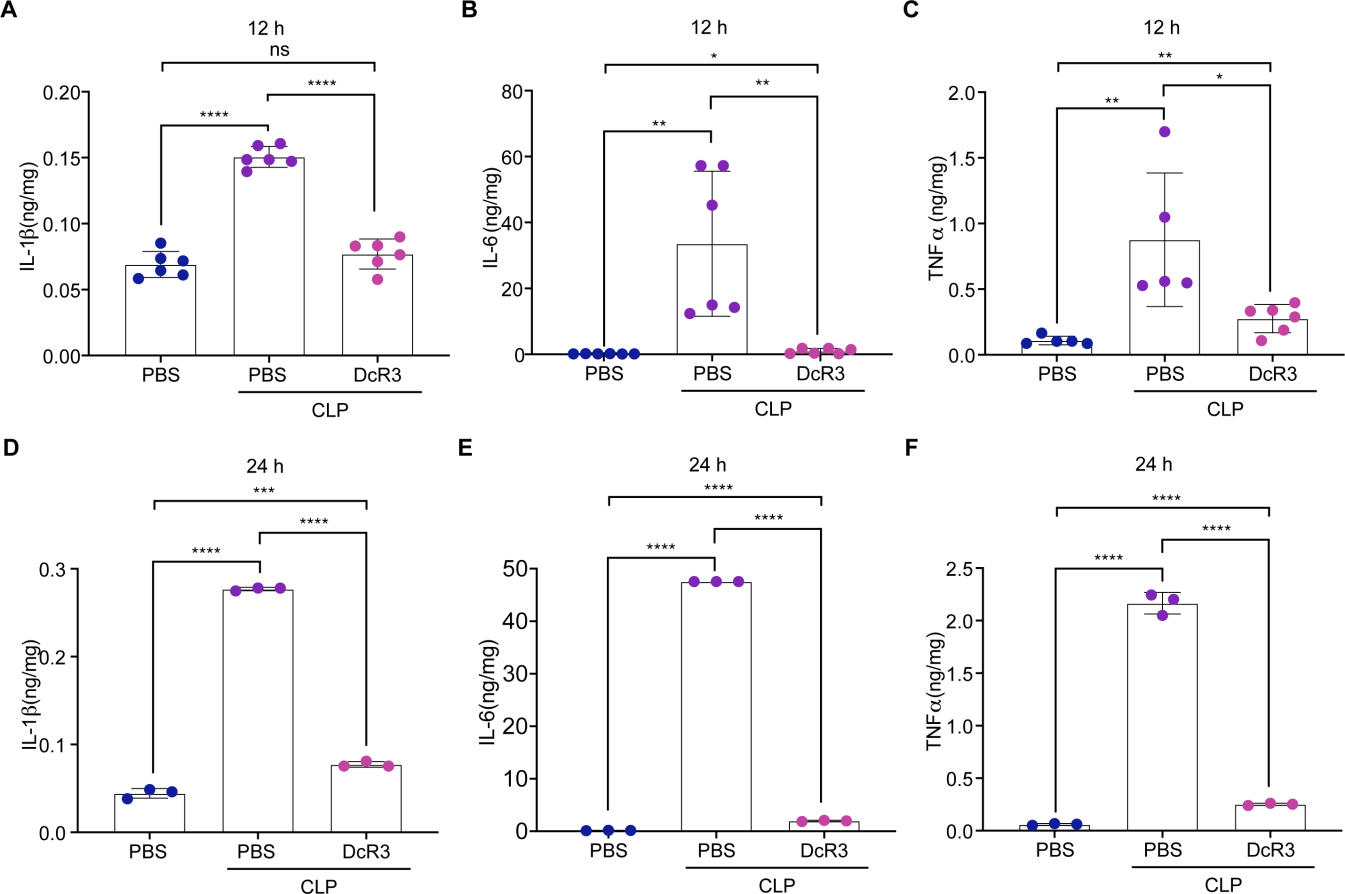
DcR3 treatment reduced the expression levels of inflammatory factors in the serum of CLP-induced septic mice. (**A–C**) DcR3 treatment in CLP-induced septic mice at 12 h. (**A**) IL-1β, (**B**) IL-6, and (**C**) TNF-α. (**D–F**) DcR3 treatment of CLP-induced septic mice at 24 h. (**D**) IL-1β, (**E**) IL-6, and (**F**) TNF-α. ANOVA and Tukey’s post hoc test were used to analyze the data. (∗) *P* < 0.05, (∗∗) *P* < 0.01, (∗∗∗) *P* < 0.001, and (∗∗∗∗) *P* < 0.0001. An independent experiment was conducted thrice to produce the results.

Next, to investigate the role of DcR3 in modulating inflammatory cytokine expression *in vivo*, we examined whether DcR3 inhibited the CLP-induced inflammatory cytokine levels in the serum, heart, liver, spleen, lung, and renal tissues (at 12 and 24 h following induction). Results of enzyme-linked immunosorbent assay (ELISA) showed that DcR3 treatment significantly reduced the CLP-induced increase in IL-1β, IL-6, and TNF-α levels in the sera at 12 and 24 h (*P* < 0.05, Fig. 3). Similar results were obtained in the heart, liver, spleen, lung, and renal tissues at 12 and 24 h (Fig. S3 and S4). Furthermore, the inhibitory effects of DcR3 on the mRNA expression of inflammatory cytokines (*IL-1*β*, IL-6, TNF-*α*, CCL2, CXCL10,* and *NLRP3*) in the heart, liver, spleen, lungs, and renal tissues following CLP induction were investigated using RT-qPCR. The DcR3-treated group showed significantly lower expression levels of inflammatory cytokines than the CLP group (*P* < 0.01, Fig. S8). These results indicate that DcR3 significantly inhibited the upregulation of inflammatory cytokines in mice with CLP-induced sepsis.

### DcR3 ameliorated CLP-induced pathology

**Fig 4:**
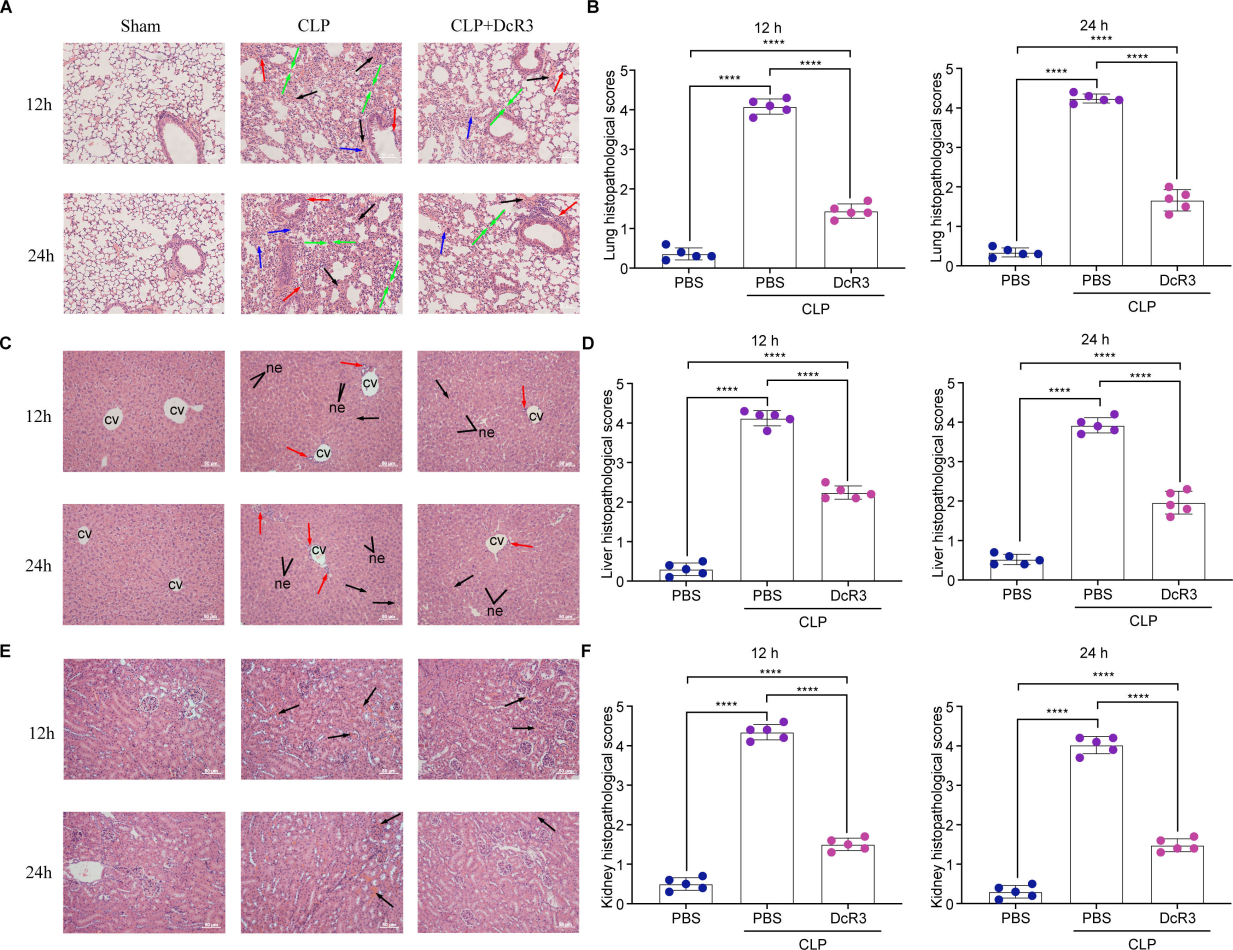
DcR3 exerts protective effects against the lung, liver, and heart tissue lesions in CLP-induced septic mice at 12 and 24 h (scale bars = 50 µm). Representative microscopic images of the (**A, B**) lung, (**C, D**) liver, and (**E, F**) renal tissues of mice with CLP-induced sepsis after hematoxylin and eosin (H&E) staining (magnification, 200×). Pathological scores of the (**B**) lung, (**D**) liver, and (**F**) renal tissues of CLP-induced septic mice. Data are expressed as mean ± standard deviation. (∗∗∗∗) *P* < 0.0001; ns, not significant (*P* > 0.05). An independent experiment was conducted five times to produce the results.

To determine whether DcR3 ameliorated CLP-induced pathological changes in the lung, liver, and renal tissues, hematoxylin and eosin (H&E) staining was performed at 12 and 24 h after DcR3 treatment. As illustrated in Fig. 4, DcR3 treatment alleviated lung injury and attenuated alveolar wall thickening, as well as hemorrhage induced by CLP in mice (*P* < 0.05, Fig. 4A and B). A similar trend was observed in the liver, with the DcR3-treated group presenting a lower level of liver injury, reduced inflammatory cells, decreased swelling, and a more regular arrangement of hepatocytes than those in the CLP group (*P* < 0.05, Fig. 4C and D). After treatment with DcR3, renal injury was diminished, the degree of glomerular rupture was reduced, and the shape of glomeruli was regular (*P* < 0.05, Fig. 4E and F). These findings suggest that DcR3 significantly ameliorated the damage caused by CLP to the lung, liver, and renal tissues of mice.

### DcR3 altered the immune cell count in mice with CLP-induced sepsis

**Fig 5:**
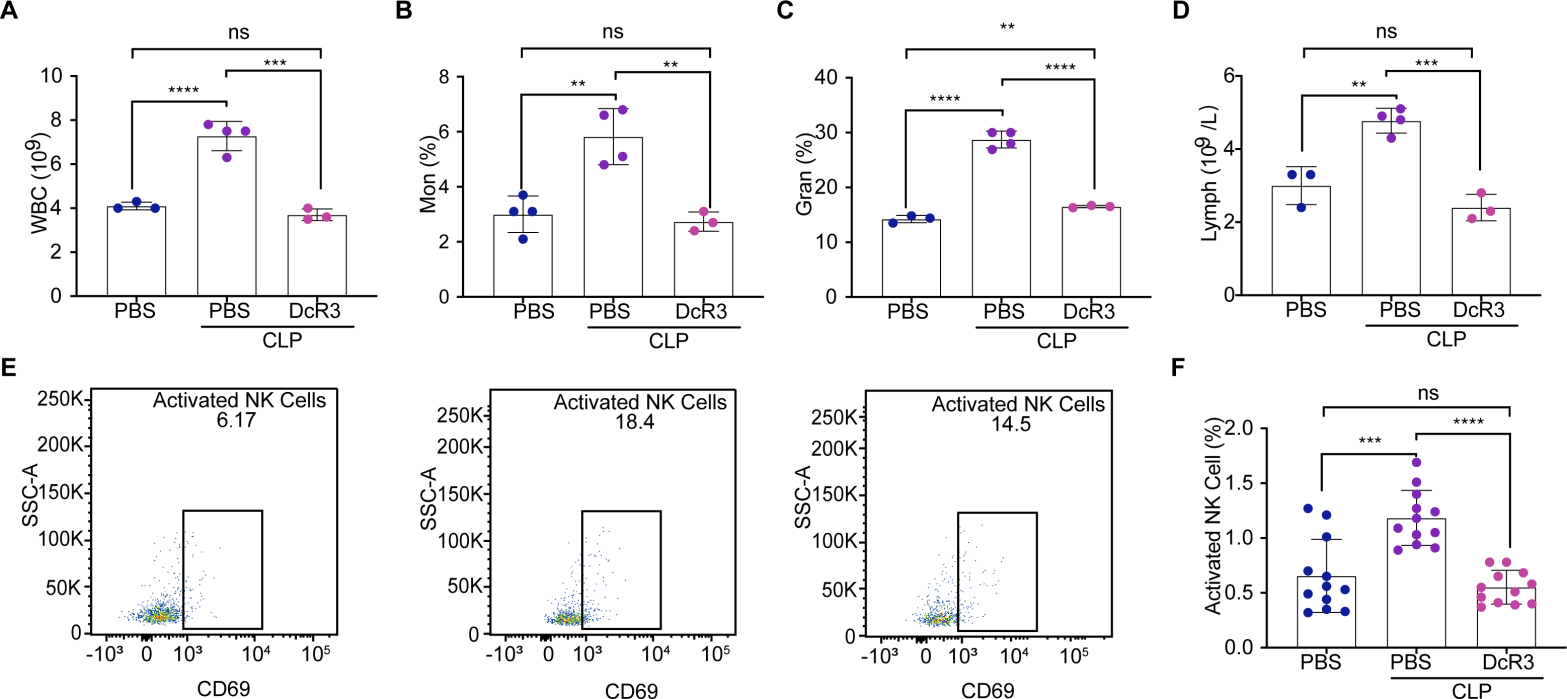
Effect of DcR3 treatment on immune cells in CLP-induced septic mice. (**A–D**) White blood cells (WBC), monocytes (Mon), granulocytes (Gran), and lymphocytes (LymPh) in peripheral blood were analyzed at 12 h after DcR3 administration in CLP-induced septic mice (*n* = 3). (**E**) Flow chart and detection markers of NK and activated NK cells using flow cytometry in CLP-induced septic mice at 12 h (*n* = 12). (**F**) Number of NK cells at 12 h. ANOVA and Tukey’s post hoc test were performed to analyze the data. (∗) *P* < 0.05, (∗∗) *P* < 0.01, (∗∗∗) *P* < 0.001, and (∗∗∗∗) *P* < 0.0001; ns, not significant (*P* > 0.05). Sample sizes are indicated in brackets.

The effect of DcR3 on the circulating immune cells was investigated by quantifying the peripheral blood of mice with CLP-induced sepsis. The white blood cell (WBC) count in the DcR3-treated group was significantly lower than that in the CLP group (*P* < 0.001) and was similar to that in the Sham group (Fig. 5A). As part of the immune response, WBCs include monocytes (Mon), granulocytes (Gran), and lymphocytes (LymPh) (21). A significant reduction in Mon, Gran, and LymPh was observed in the DcR3-treated group compared to the CLP group (*P* < 0.01, Fig. 5B through D). Using flow cytometry gating strategies (Fig. S9), we determined whether DcR3 treatment improved immune function in mice with CLP-induced sepsis. At 12 h after DcR3 administration, the number of activated NK cells was significantly lower in the DcR3 + CLP group than that in the CLP group (*P* < 0.0001, Fig. 5E and F). However, no significant change in B220, B, CD4^+^ T, CD8^+^ T, activated CD4^+^ T, activated CD8^+^ T, Treg, and activated Treg cells was observed at 24 h in peripheral blood of CLP-induced sepsis mice (*P* > 0.05, Fig. S10 through S14). These findings suggest that DcR3 alters activated NK cells in mice with CLP-induced sepsis.

### Protective effect of DcR3 on intestinal barrier in CLP-induced sepsis mice

**Fig 6:**
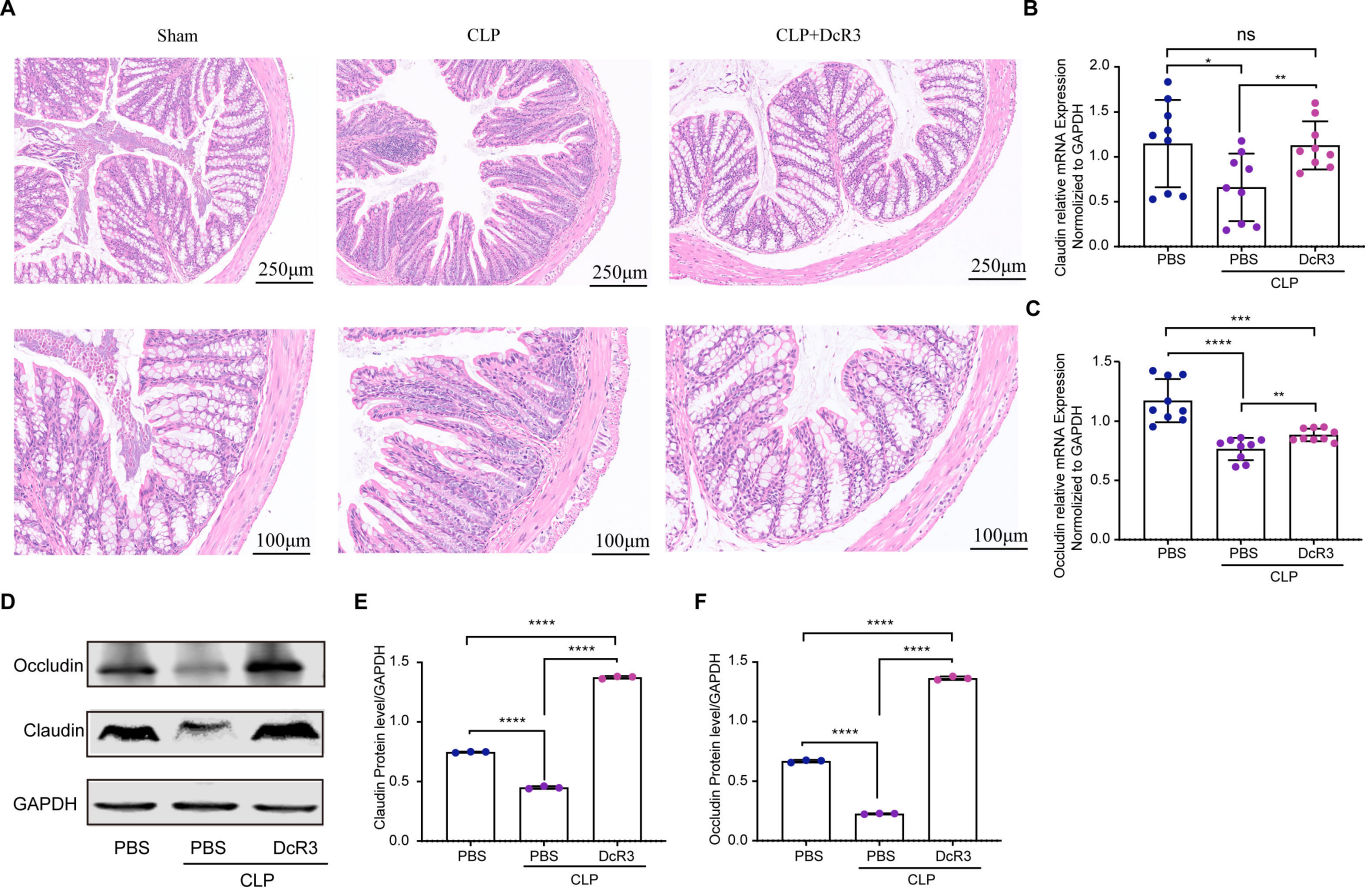
The role of DcR3 in restoring intestinal morphology and barrier function in CLP-induced sepsis mice. (**A**) Representative HE-stained histological sections. The scale bars are shown in the figure. (**B, C**) Relative mRNA expression of *Cldn1* and *Ocln*. (**D**) Western blotting to measure the protein expression levels of Claudin-1 and Occludin in colon tissue after 12 h of DcR3 (1 mg/kg) treatment. (**E, F**) Densitometric analysis of bands via ImageJ software. Details the relative protein levels of Claudin-1 and Occludin. Data are expressed as mean ± standard deviation and analyzed using ANOVA and Tukey’s post hoc test. Statistical significance is denoted by (∗) *P* < 0.05, (∗∗) *P* < 0.01, (∗∗∗) *P* < 0.001, and (∗∗∗∗) *P* < 0.0001; ns, not significant (*P* > 0.05).

The role of DcR3 in protecting the intestinal barrier in CLP-induced sepsis mice was investigated. As shown in Fig. 6A, colonic tissue in the Sham group remained normal, with orderly crypts and intact epithelial cells, as compared with the severe tissue damage and increased permeability observed in the CLP group, which were notably mitigated following DcR3 treatment.

The study underscores the crucial function of the intestinal barrier’s physical structure, especially the role of tight junction proteins (Occludin and Claudin-1). Disruption in the levels of these proteins, as observed in the CLP group, was linked to heightened intestinal permeability and inflammation (22). DcR3 administration reversed these effects, as evidenced in Fig. 6B through F by significantly upregulating both the mRNA and protein levels of these proteins (*P* < 0.01), showcasing a dual protective action. This investigation, thus, revealed DcR3’s dual-action mechanism, effectively counteracting sepsis-induced intestinal barrier dysfunction at both the mRNA and protein expression levels.

### DcR3 regulated intestinal microbiota homeostasis in mice with CLP-induced sepsis

**Fig 7:**
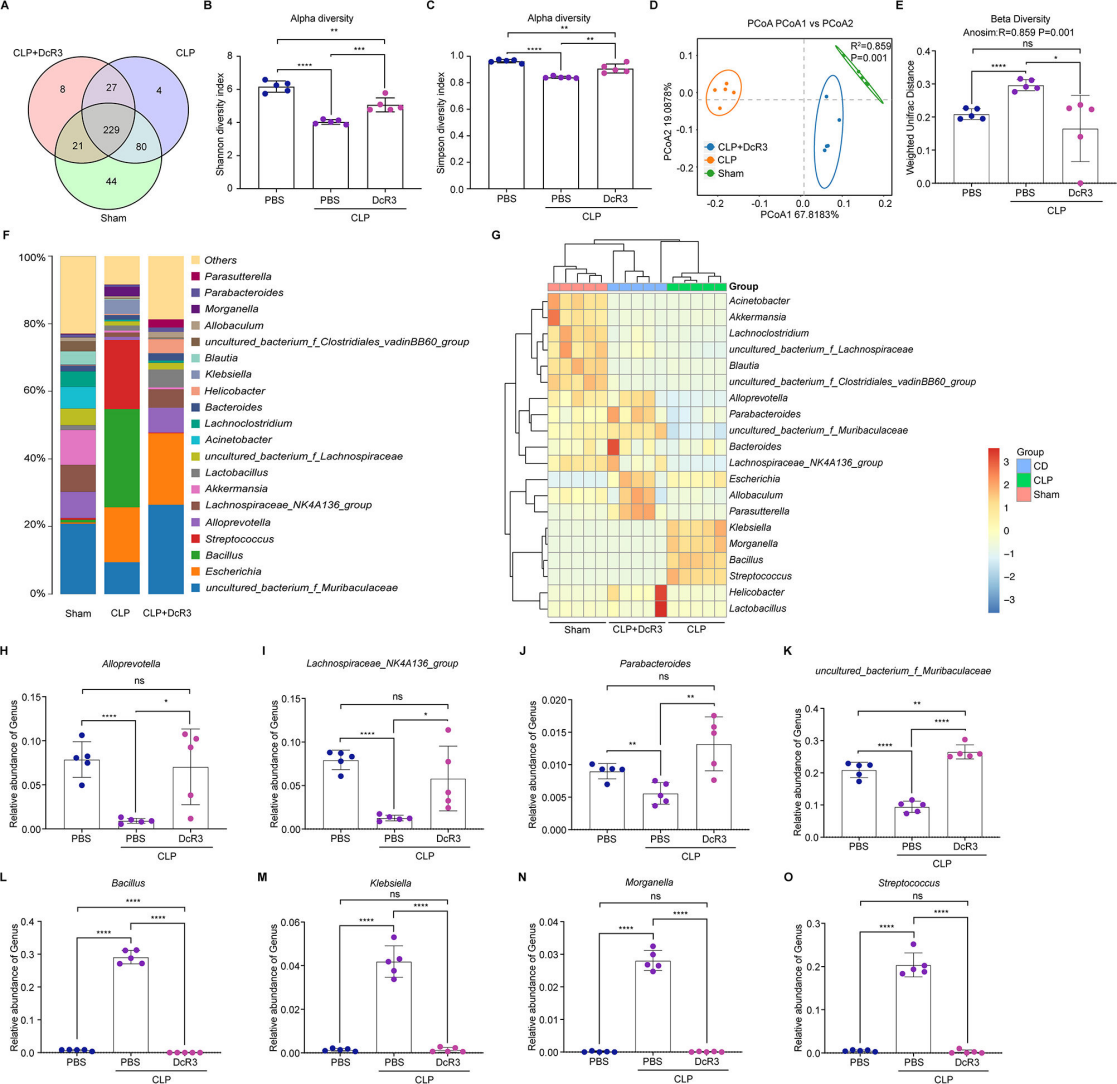
DcR3 remodels the intestinal microbiota homeostasis in CLP-induced septic mice. (**A**) Venn diagram showing OTUs of intestinal microorganisms. (**B, C**) Alpha diversity analysis of intestinal microbiota at the OTU level. (**B**) Shannon and (**C**) Simpson indices. (**D, E**) Beta diversity analysis of the intestinal microbiota at the OTU level. (**D**) Beta diversity PCoA plots based on weighted UniFrac Adonis analysis in distinct groups. (**E**) Beta diversity based on weighted UniFrac ANOSIM analysis in distinct groups. (**F**) Histogram showing species distribution at the genus level. (**G**) Heat map analysis of the relative abundance of intestinal microorganisms in the distinct groups at the genus level. Relative abundance of (**H**) *Alloprevotella*, (**I**) *Lachnospiraceae_NK4A136_group*, (**J**) *Parabacteroides*, (**K**) *uncultured_bacterium_f_Muribaculaceae*, (**L**) *Bacillus*, (**M**) *Klebsiella*, (**N**) *Morganella*, and (**O**) *Streptococcus*. The value corresponding to the heat map is the *Z*-value obtained after the standardization of the relative abundance of species in each row. ANOVA and Tukey’s post hoc test were performed to analyze the data. (∗) *P* < 0.05, (∗∗) *P* < 0.01, (∗∗∗) *P* < 0.001, and (∗∗∗∗) *P* < 0.0001; ns, not significant (*P* > 0.05). An independent experiment was conducted five to produce the results.

As shown in Fig. 7A, the number of operational taxonomic units (OTUs) unique to the Sham, CLP, and DcR3-treated groups were 44, 4, and 8, respectively. The analysis of alpha diversity indices showed that the Shannon and Simpson indices in the DcR3-treated group were significantly different from those in the CLP group (*P* < 0.01, Fig. 7B and C). Principal coordinate analysis was performed, and a significant difference in gut microbial communities was observed among the three groups (Fig. 7D). Next, similar results were obtained for beta diversity analysis, which was based on weighted UniFrac distances (Fig. 7E).

To further investigate the variation in microbiota structure, the relative abundances at the phylum, family, and genus levels were analyzed (Fig. 7F; Fig. S15A and B). At the genus level, the mouse gut microbiota was dominated by *uncultured_bacterium_Muribaculaceae*, *Escherichia*, *Bacillus*, *Streptococcus*, *Alloprevotella*, *Lachnospiraceae* NK4A136 group, *Lactobacillus*, and *Klebsiella* (Fig. 7F).

As illustrated in Fig. 7G through O, compared to the CLP group, the abundance of *Alloprevotella*, *Lachnospiraceae* NK4A136 group, *Parabacteroides*, *uncultured_bacterium_Lachnospiraceae*, and *uncultured_bacterium_Muribaculaceae* increased significantly in the DcR3-treated group (*P* < 0.05). Conversely, DcR3 treatment significantly reduced the abundance of *Bacillus*, *Klebsiella*, *Morganella*, and *Streptococcus* in the DcR3-treated group compared to the CLP group (*P* > 0.05). The different microbial groups in mice were analyzed using the linear discriminant analysis effect size (LEfSe) assay (Fig. S15C). Using linear discriminant analysis (LDA) values > 4 as the screening criterion, we detected a total of 50 different species (Fig. S15D). Therefore, DcR3 effectively reduces pathogenic bacterial abundance, increases beneficial bacterial abundance, and significantly ameliorates gut dysbiosis in septic mice.

### DcR3 promoted short-chain fatty acids production in mice with CLP-induced sepsis

**Fig 8:**
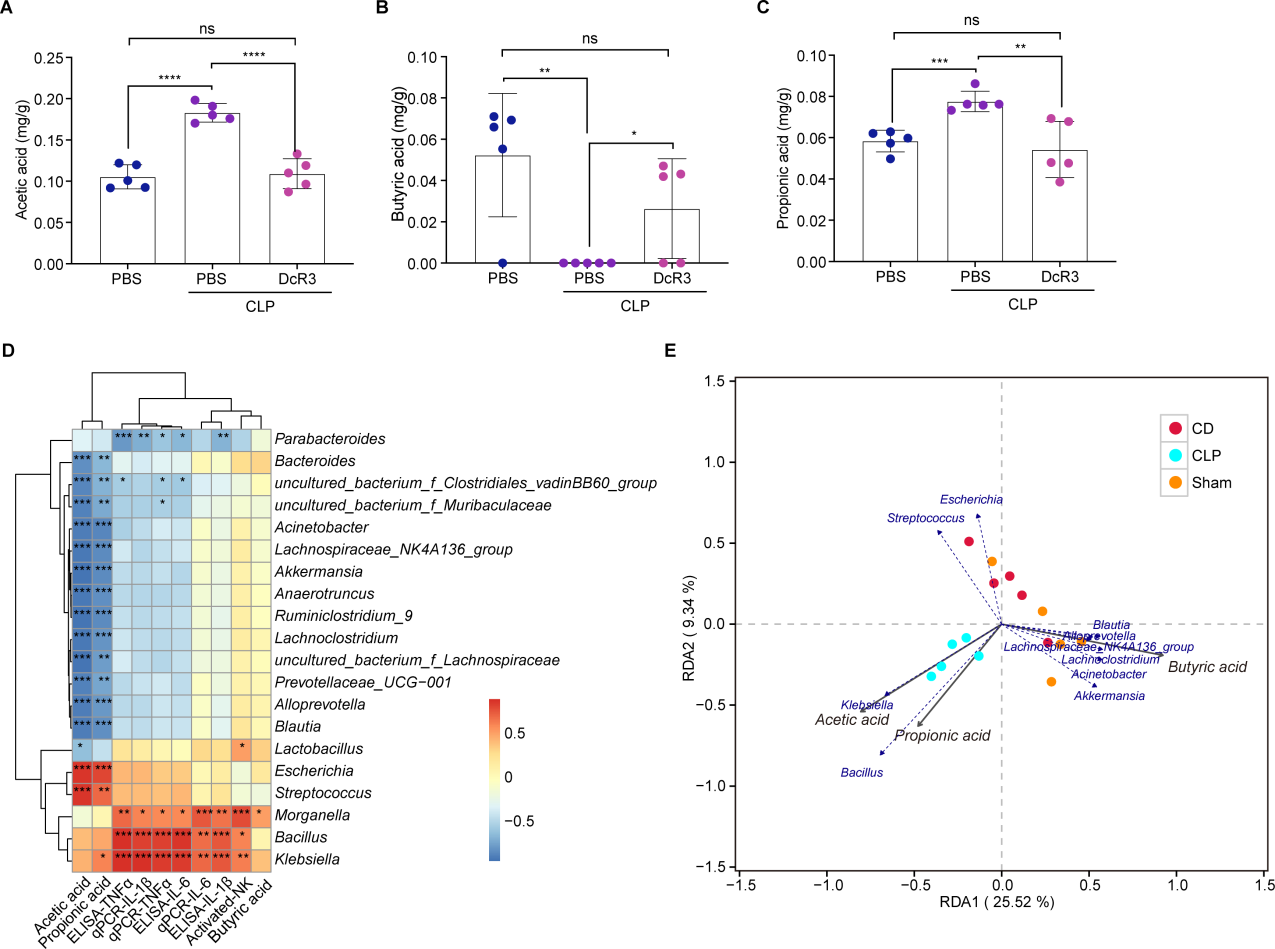
Correlation between intestinal microbiota and environmental factors in CLP-induced septic mice after DcR3 treatment. Effects of DcR3 on (**A**) acetic, (**B**) butyric, and (**C**) propionic acid levels produced by intestinal microorganisms in CLP-induced septic mice (*n* = 5). (**D**) Spearman’s rank correlation heat map among bacterial genera, levels of SCFAs, and serum, as well as levels of IL-6, IL-1β, and TNF-α in the lungs of WT mice and CLP-induced septic mice. (**E**) Redundancy analysis/canonical correspondence analysis between bacterial genera and levels of SCFAs in CLP-induced septic mice. Data are expressed as mean ± standard deviation. ANOVA and Tukey’s post hoc test were performed to analyze the data. (∗) *P* < 0.05, (∗∗) *P* < 0.01, (∗∗∗) *P* < 0.001, and (∗∗∗∗) *P* < 0.0001; ns, not significant (*P* > 0.05). An independent experiment was conducted five to produce the results.

As major metabolites of intestinal microbiota, short-chain fatty acids (SCFAs) play a crucial role in inflammation and immunity. Following DcR3 treatment, acetic and propionic acid significantly decreased in the DcR3-treated group compared to those in the CLP group (*P* < 0.01), whereas a significant increase in butyric acid was observed after treatment (*P* < 0.05, Fig. 8A through C). Next, a correlation analysis was conducted between proinflammatory cytokines, SCFAs, and intestinal microbiota. Results showed that *Escherichia* and *Streptococcus* were significantly positively correlated with acetic and propionic acids, whereas *Blautia*, *Alloprevotella*, *uncultured_bacterium_Muribaculaceae*, *Alloprevotella*, *Lachnospiraceae* NK4A136 group, and *Akkermansia* were significantly negatively correlated with the two SCFAs. Furthermore, the expression of IL-1β, IL-6, and TNF-α in the serum and lung tissues was positively correlated with the abundance of *Klebsiella*, *Morganella*, and *Bacillus* (Fig. 8D). Finally, the levels of acetic and propionic acids were positively correlated with the abundance of *Klebsiella* and *Bacillus* (Fig. 8E).

### DcR3 inhibited CLP-induced inflammatory response and apoptosis

**Fig 9:**
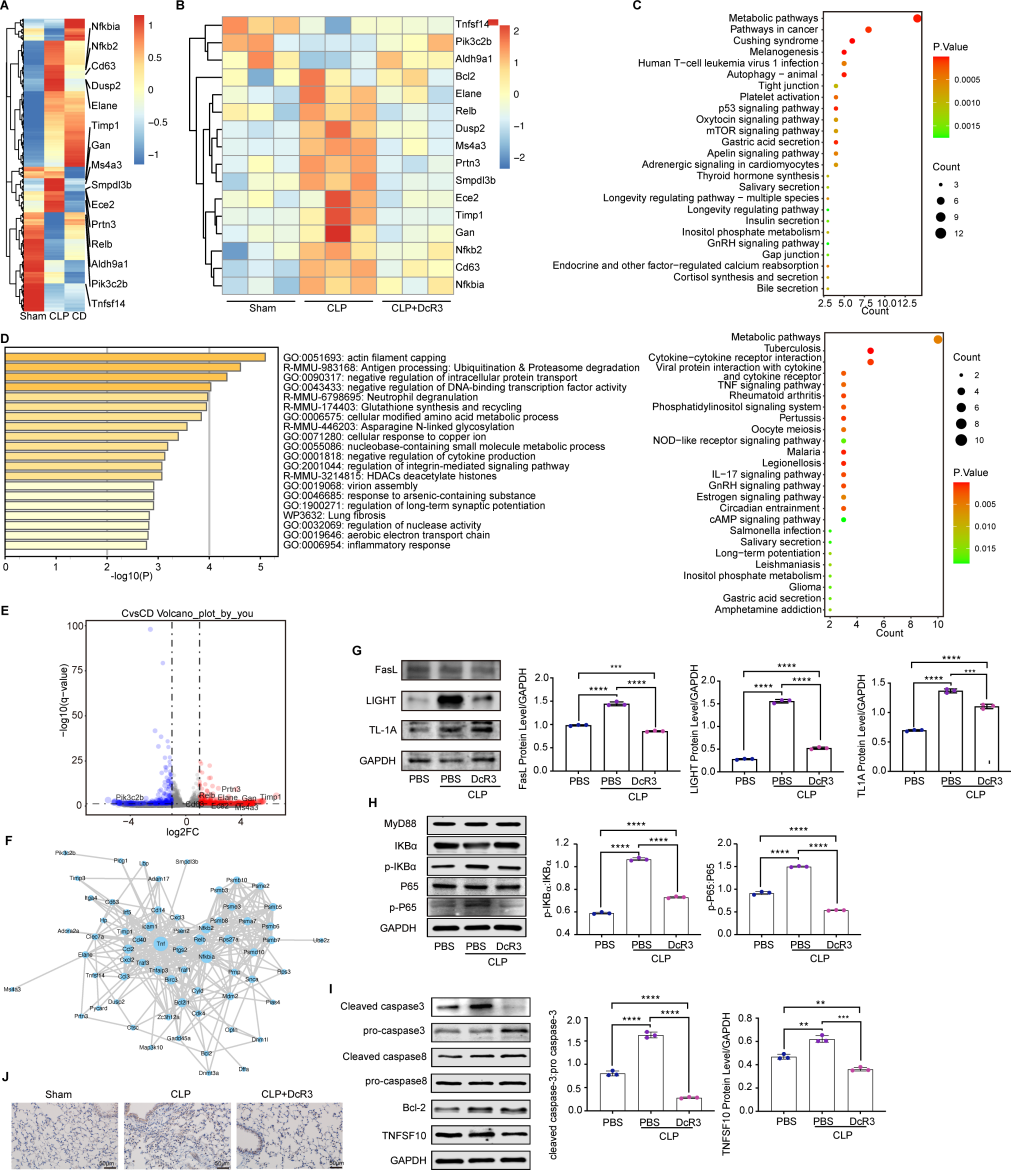
Impact of DcR3 on NF-κB and caspase pathways in septic mice induced by CLP. (**A–F**) Analysis of gene profiles in septic mice post-CLP with DcR3 treatment. (**A**) Clustered heatmap detailing gene expression in Sham, CLP and DcR3-treated septic mice. (**B**) DEG heatmap in Sham, CLP and DcR3-treated septic mice. (**C**) KEGG analysis of DEGs in Sham, CLP and DcR3-treated septic mice (Sham vs CLP and CLP vs CLP + DcR3). (**D**) GO enrichment of DEGs (*P* < 0.05). (**E**) Volcano plot showcasing DEGs post-DcR3 treatment compared with those in CLP-induced septic mice (CLP vs CLP + DcR3; downregulated genes, blue; upregulated genes, red; insignificantly altered genes, black). (**F**) Identification of protein interaction networks among TNF, NF-κB, and Bcl2 using the STRING database. (**G**) Effect of DcR3 treatment on FasL, LIGHT, and TL1A in CLP-induced septic mice. (**H**) Western blotting to assess MyD88, NF-κB (P65) and p-NF-κB (P-P65) protein levels in lung homogenates after 12 h of DcR3 (1 mg/kg) treatment. (**I**) Western blotting to measure the protein expression levels of cleaved caspase 3, pro-caspase 3, cleaved caspase 8, pro-caspase 8, Bcl-2, and TNFSF10 in lung homogenates after 12 h of DcR3 (1 mg/kg) treatment. (**G–I**) Densitometric analysis of bands via ImageJ software. (**J**) p-NF-κB (P-P65) levels in lung tissues determined through immunohistochemistry. Scale bar  =  50  µm. Data are presented as mean ± standard deviation (*n* = 3). ANOVA and Tukey’s post hoc test was performed to analyze the data. (∗∗) *P* < 0.01, (∗∗∗) *P* < 0.001, and (∗∗∗∗) *P* < 0.0001.

A comprehensive whole-genome RNA-sequencing was conducted to elucidate DcR3’s mechanistic role in sepsis defense. Through heatmaps (Fig. 9A and B), we showed changes in gene expression and identified key differentially expressed genes (DEGs). Notably, DcR3 reduced activity in critical genes across several pathways, including the NF-κB (*NFKBIA, NFKB2*), TNF (*TNFSF14*), MAPK/ERK (*DUSP2*), phosphatidylinositol system (*PIK3C2B*), and apoptosis-related pathways (*BCL2*).

Further analysis using Gene Ontology (GO) and Kyoto Encyclopedia of Genes and Genomes (KEGG) on DEGs highlighted DcR3’s regulatory effects. CLP notably enhanced cytokine interactions and TNF, phosphatidylinositol, NOD-like receptor, and IL-17 signaling pathways. Conversely, DcR3 effectively dampened inflammatory responses, disrupted tight junctions, and mitigated apoptotic processes (Fig. 9C and D). Integration of volcano plot and STRING analyses provided insights into protein interactions involving TNF, NF-κB, Bcl2, Relb, Rps27a, Cxcl2, and Cd40 (Fig. 9E and F). DcR3 exhibits a strong affinity for competitively binding to FasL, LIGHT, and TL1A, effectively inhibiting downstream signaling pathways triggered by CLP (*P* < 0.001, Fig. 9G). Lung tissue immunoblotting for investigating DcR3’s impact on CLP-induced sepsis revealed key inflammatory and apoptotic markers. Significant NF-κB activation marked by increased phosphorylation of NF-κB (p65) and IKBα in CLP was notably attenuated by DcR3 (*P* < 0.001, Fig. 9H). Additionally, a marked decrease in cleaved caspase-3 ratios post-DcR3 treatment highlighted its role in reducing apoptosis (*P* < 0.001; Fig. 9I). Immunohistochemical analysis further confirmed DcR3’s inhibition of NF-κB (p65) phosphorylation in the lung tissues of septic mice (Fig. 9J). Collectively, these results pinpoint NFKBIA, NFKB2, TNFSF14, DUSP2, and PIK3C2B as key mediators of DcR3’s broad-spectrum effects in CLP-induced sepsis. The DcR3’s protective mechanism involves curtailing the inflammatory response and apoptotic activation triggered by CLP, delineating a novel therapeutic pathway in sepsis management.

## DISCUSSION

To the best of our knowledge, this is the first study to report the therapeutic effect of DcR3 constructed without the Fc fragment on CLP-induced sepsis in mice. Our *in vitro* and *in vivo* results indicate that DcR3 and DcR3.Fc had similar anti-inflammatory properties. DcR3 improved the survival rate of mice with CLP-induced sepsis (from 25% to 60%). Histopathological analyses revealed that DcR3 ameliorated CLP-induced lung and liver damage in mice. Furthermore, DcR3 significantly inhibited the excessive activation of immune cells in peripheral blood of mice with CLP-induced sepsis and considerably reduced the number of activated NK induced by CLP. This study’s novelty lies in its demonstration of DcR3’s dual-action at both the mRNA and protein levels, providing an innovative therapeutic approach in sepsis-induced intestinal barrier dysfunction. After treatment with DcR3, mice with CLP-induced sepsis showed a high abundance of probiotics and a low abundance of pathogens or opportunistic pathogens. Alternatively, acetic and propionic acids were significantly reduced by DcR3. *Parabacteroides* abundance was highly correlated with the reduced expression levels of inflammation-related factors. Furthermore, DcR3t inhibited CLP-induced IκBα phosphorylation. Thus, we identified the mechanism underlying systematic inflammatory response inhibition and its potential relationship with gut microbiota alteration.

During sepsis, monocytes and neutrophils secrete numerous inflammatory cytokines and produce a “cytokine storm” by promoting lymphocyte proliferation, thus causing cytotoxicity (23, 24). The “cytokine storm” is markedly improved following apoplectic cell administration (25). Attenuation of inflammation and apoptosis has become a critical component in treating sepsis (26, 27). DcR3 can competitively bind to FasL, LIGHT, and TL1A through its decoy function, thereby blocking the original biological activity of the ligands and the downstream signal transduction, which ultimately leads to anti-inflammatory effects (14, 28). DcR3.Fc inhibits the TL1A-induced activation of NF-κB (29), and DcR3 can reduce the expression levels of inflammatory markers by neutralizing FasL. DcR3 also exerts “non-decoy” functions through direct anti-inflammatory signaling (28). Lung cytokine and neutrophil infiltration induced by FasL were significantly reduced by exogenous DcR3 analog injections (30). Overactivated T cells and imbalanced TNF superfamily signaling can impair bacterial clearance in the lungs. As a result of treatment with the DcR3 analog, T cell activation can be reduced, neutrophil function can be restored, and bacterial clearance can be enhanced (31). These findings led us to use recombinant DcR3.Fc protein *in vitro* and administer it to mice with CLP-induced sepsis. A significant improvement in survival rate, reduced inflammation, improved organ lesions, and inhibited lymphocyte proliferation were observed (15). The Fc domain of the fusion protein binds to Fc receptors on the surface of immune cells, thereby inducing excessive immune activation, such as antibody-dependent enhancement (32). Due to adverse reactions caused by the Fc fragment, use of the endogenous DcR3 has become the preferred option, and a bioactive agent with a more appropriate form is urgently needed (16, 17). Endogenous DcR3 production is enhanced in the human body under pathological conditions. However, endogenous DcR3 may not improve sepsis *in vivo* due to the limited amount of Dcr3 induced to confer an overwhelming advantage (19). Healthy organisms maintain homeostasis by maintaining a dynamic balance between gut-mediated immunity and the host’s inflammatory response (33). For decades, the gut has been considered a major factor in sepsis pathogenesis. Sepsis alters the gut microbiome composition and leads to organ failure through microbiota–immune interactions. Changes in bacterial abundance, translocation of gut bacteria, and imbalance in the gut microbiota occur as a result of disruption of homeostasis (34). For the first time, we demonstrated that DcR3 treatment significantly increased the abundance of *Alloprevotella*, *Lachnospiraceae* NK4A136 group, *Parabacteroides*, and *uncultured_bacterium_Muribaculaceae*, and decreased the abundance of *Bacillus*, *Klebsiella*, *Morganella*, and *Streptococcus*. Our results showed a negative correlation between *Parabacteroides* and the expression of inflammatory cytokines, including IL-1β, IL-6, and TNF-α. Members of *Bacteroidetes,* such as *Parabacteroides,* have been suggested to benefit the host by preventing the infection with potential pathogens that may colonize and infect the gut (35). *Parabacteroides* confers protection from seizure, obesity, multiple sclerosis, tumors, and metabolic dysfunctions (36, 37). This genus is beneficial to the host in several ways: it decreases inflammation, regulates immunity, reduces carbon metabolism, and secretes SCFAs. Overall, our results suggest that the role of DcR3 in regulating inflammation may be closely related to the changes in intestinal flora during sepsis although the logical associations (causality) among the three remains unclear. Considering that inflammation and microbial alterations in sepsis constitute a vicious cycle (38), exploring the potential relationship between the effects of new drugs on inflammation and gut microbiota is of great clinical importance.

This study has certain limitations that need to be addressed. First, initiating our intervention merely 1 h post-CLP may be considered precocious in clinical practice. Recognizing this limitation in translating our findings to human trials is pivotal. Addressing this is of utmost importance, given the gamut of challenges, including the arduous definition of “time zero” in sepsis, to ensure treatment safety, accommodate patient heterogeneity, and flexibly adapt trial designs. These factors underscore the compelling need for further profound research and adaptive measures when extrapolating experimental results to clinical practice. Second, owing to the opulent and intricate structure of the gut microbiota, the interaction between DcR3 and sepsis-associated gut microbiota remains shrouded in ambiguity. Hence, employing fecal transplants and germ-free mice becomes imperative for elucidating this relationship.

### Conclusion

DcR3 exerted a protective effect against CLP-induced sepsis in mice by reducing the levels of inflammatory cytokines. *In vitro* and *in vivo* experiments revealed that the protective effect of DcR3 against sepsis was associated with its anti-inflammatory activities. These findings provide valuable insights into the molecular mechanisms underlying the protective effects of DcR3 against sepsis, thus paving the way for future clinical studies.

## MATERIALS AND METHODS

### Chemicals and reagents

Recombinant chimeric human DcR3-Fc protein (DcR3.Fc) was purchased from R&D Systems (Minneapolis, MN, USA). The Green qPCR SuperMix reverse transcription kit and bicinchoninic acid (BCA) protein concentration kit were purchased from TransGen (Beijing, China). The ELISA kits were purchased from R&D Systems. The antibodies used in the study and their manufacturers are listed in Table 1.

**TABLE 1:** Antibodies used in the study

Antibody	Manufacturer	Identifier
GAPDH	Proteintech	Cat#60004-1-Ig
NF-κB	Cell Signaling Technology	Cat#6956
IκBα	Abmart	Cat#T55026
p-IκBα	ABclonal Technology	Cat#AP0999
MyD88	Abmart	Cat#TA5195
TNFSF10	Proteintech	Cat#27064-1-AP
Bcl-2	Abmart	Cat#126BA
pro-caspase 8	Proteintech	Cat#13423-1-AP
Cleaved caspase 8	Cell Signaling Technology	Cat#98134S
pro-caspase 3	Proteintech	Cat#19677-1-AP
Cleaved caspase 3	Cell Signaling Technology	Cat#9664S
CD45 BUV395-A	Becton, Dickinson and Company	Cat#564279
CD45R BB515-A	Becton, Dickinson and Company	Cat#553088
CD3 APC-Alexa 700A	Becton, Dickinson and Company	Cat#561388
CD8 BUV805-A	Becton, Dickinson and Company	Cat#612898
CD25 BB700-A	Becton, Dickinson and Company	Cat#567482
NK1-1 PE-CF594-A	Becton, Dickinson and Company	Cat#562864
CD69 BV711-A	Becton, Dickinson and Company	Cat#740664
CD4 BUV496-A	Becton, Dickinson and Company	Cat#612952
CD120b BV421-A	Becton, Dickinson and Company	Cat#564088
CD127 PE-Cy7A	Becton, Dickinson and Company	Cat#560733
CD20 PE-BYG568-A	BioLegend	Cat#152106
Occludin	Proteintech	Cat#27260-1-AP
Claudin-1	Proteintech	Cat# 13050-1-AP

### Recombinant DcR3 protein

To obtain the recombinant DcR3 protein, cDNA encoding human DcR3 (1–828) was inserted into the pSmart-I vector (Novagen, Germany) containing an N-terminal 6×His tag. The expression and purification method were performed as described previously (39).

### Cell lines and culture conditions

The anti-inflammatory properties of DcR3 and DcR3-Fc were investigated *in vitro* using murine macrophages (RAW 264.7) obtained from the American Type Culture Collection (Manassas, VA, USA). The cells were cultured in Dulbecco’s modified Eagle’s medium (Gibco, Grand Island, NY, USA), containing 10% (vol/vol) fetal bovine serum and 1% (vol/vol) penicillin/streptomycin (Gibco), and incubated in 5% CO_2_ at 37°C.

### Cell viability assay

Cell viability was evaluated using the Cell Counting Kit-8 (CCK-8; Beyotime Biotechnology, Beijing, China), as previously described (40).

### Animals

An equal number of male and female specific pathogen-free C57BL/6 mice at 8–10 weeks old were purchased from Wu’s Animal Center and reared at the Experimental Animal Center of Fujian Normal University. The temperature was maintained between 23 and 25°C, with humidity ranging from 40% to 60% in a 12 h light/dark cycle. Mice had free access to food and water. Male (20–22 g) and female mice (18–20 g) were grouped randomly.

### LPS-induced sepsis mouse model

LPS was used to induce sepsis as previously described (40). LPS used in our study was obtained from *Escherichia coli* 0111:B4 cells (Cell Signaling Technology, Beverley, MA, USA). The Sham group received an equivalent volume of phosphate-buffered saline (PBS) (0.0067M; pH 7.4, HyClone, GE Healthcare Life Sciences, UT, USA). The mice were intraperitoneally (ip) injected with FX 30 min prior to the ip administration of a lethal dose of LPS (20 mg/kg) or PBS. Additionally, all mice were fasted for 12 h preoperatively but had access to water.

### CLP model of sepsis

Sepsis was induced using the CLP model as previously described (41). Mice were anesthetized with an ip injection of 0.3% pentobarbital sodium. The mice were positioned on the operating table, and the lower left abdomen area was shaved using an electric hair shaver. The shaved skin was then sterilized with betadine solution and 75% alcohol. A longitudinal incision was carefully made in the appropriate abdominal area, followed by a 1 cm incision through the layers of the abdominal wall, including the cortical and muscle layers, to expose the cecum. The cecum was gently exteriorized using forceps, and a ligature was applied to approximately two-thirds of the cecum near the base of the ileal valve using a sterile 4.0 surgical suture. A small puncture was made in the ligated area using a sterile 5-mL syringe needle, and a small amount of fecal material was extruded.

The ligated and punctured cecum was carefully returned to the abdominal cavity, and the muscle and skin layers were sequentially sutured using a sterile 4.0 surgical suture. Subcutaneous administration of 1 mL of preheated 0.9% normal saline at 37 ℃ was performed on the back for fluid resuscitation. The mice were placed on a heating pad until they fully recovered. The control (Sham) group underwent a laparotomy procedure without cecal ligation or puncture.

### Experimental protocol

Mice were randomly grouped into three: Sham, CLP model, and DcR3 treatment groups (1.0 mg/kg/d). Mice were treated with DcR3 (1.0 mg/kg/d) 1 h after sepsis induction. Animal survival was examined at 6 h intervals after drug administration. Sepsis severity was assessed using the MSS (42). Rectal temperature was recorded every 4 h for 48 h using an intelligent digital thermometer (TH212; Beijing Zhongjiao Building Instrument Technology Development Co., Ltd.). Mice were anesthetized via intraperitoneal injection of pentobarbital sodium salt. Then, blood, fecal, ascite, and tissue samples were collected as previously described (43).

### Quantitative reverse transcription PCR (RT-qPCR)

Total RNA was isolated using TRIzol (Takara, Tokyo, Japan). RT-qPCR was performed as previously described (41). RT-qPCR was performed using specific primers (Table 2), with a housekeeping gene (*Gapdh*) used for normalization. The mRNA levels were measured in relative units on the *y*-axis, calculated as fold changes compared to those in the PBS control group, employing the delta-delta cycle threshold (Ct) (ΔΔCt) method. PCR for each gene was repeated three times. The average Ct value was used for gene expression stability assessment.

**TABLE 2:** Sequences of primers used for qRT-PCR^*a*^

Primer	Sequence (5′–3′)
GAPDH	F: AGAGTGTTTCCTCGTCCCG
GAPDH	R: GATGGCAACAATCTCCACTTT
IL-6	F: CTTGGGACTGATGCTGGTG
IL-6	R: TCATTTCCACGATTTCCCA
IL-1β	F: TCATTGTGGCTGTGGAGAAG
IL-1β	R: TCATCTCGGAGCCTGTAGTG
TNF-α	F: GCCTCCCTCTCATCAGTTCTA
TNF-α	R: GGCAGCCTTGTCCCTTGA
CCL2	F: GCTCAAGAGAGAGGTCTGTGCT
CCL2	R: CTACAGAAGTGCTTGAGGTGGT
NLRP3	F: CAACAGTCGCTACACGCAG
NLRP3	R: GTCCTCGGGCTCAAACAG
Occludin	F: CCCCAATGTTGAAGAGTGGGTTA
Occludin	R: CACACTCAAGGTCAGAGGAATCT
Claudin-1	F: TAATTGGCATCCTGCTGGGG
Claudin-1	R: CTGGCCAAATTCATACCTGGC

^*a*^ GAPDH: glyceraldehyde 3-phosphate dehydrogenase; IL: interleukin; TNF: tumor necrosis factor; F: forward, R: reverse.

### ELISA

The levels of IFN-β, IL-1β, IL-6, and TNF-α were assessed using ELISA, following the manufacturer’s instructions (IFN-β: 424001, Thermo Fisher Scientific; IL-6: SM6000B, IL-1β: SMLB00C, TNF-α: SMTA00B, R&D Systems, MN, USA).

### Western blotting

Western blotting was performed as previously described (43), including protein extraction, electrophoresis, membrane transfer, antibody incubation, washing, secondary antibody incubation, and chemiluminescent visualization. Protein bands were quantified and expressed as fold changes relative to controls. The antibodies used are listed in Table 1.

### Histopathological examination

Hematoxylin and eosin (H&E) staining was conducted according to established procedures (44). Lung tissues were rinsed with sterile normal saline and then fixed in 4% paraformaldehyde for 24 h. Subsequently, the specimens were subjected to a series of procedures, including dehydration, slicing into 4 µm sections, and mounting onto slides. The slides underwent deparaffinization at 60°C, followed by rehydration, and were then stained with H&E (Sigma). Excess stain was removed by rinsing with ethanol and xylene, and coverslips were applied. The assessment of tissue injury and the degree of necrosis was performed using the injury grading system, as originally proposed (grades 0–4) (45).

### Hematological parameters

The levels of peripheral blood cells, red blood cells, lymphocytes, monocytes, granulocytes, platelet hematocrit, and platelets were measured using an automated animal blood cell analyzer (Mindray Blood cells-2800VET, Mindray Animal Care, Shenzhen, China) according to the manufacturer’s instructions.

### Immunohistochemical assay

The immunohistochemical assay was performed as previously described (44). Paraffin was removed from sections using ethyl alcohol and ultrapure water, and antigens were unmasked in a microwave oven. Sections were incubated with 3% hydrogen peroxide to inhibit endogenous enzymes and with blocking solution to block nonspecific antigens. Next, sections were incubated with an anti-NF-κB (p65) antibody at 4°C overnight, followed by a secondary antibody for 1 h. Subsequently, 3,3′-diaminobenzidine tetrahydrochloride hydrate and hematoxylin staining solution were used to stain the sections. A semi-quantitative analysis was performed using the ImageJ software (NIH, MD, USA).

### Flow cytometry

Flow cytometry was performed according to a previously described method (44). The antibodies used are listed in Table 1. Red blood cells were removed using a red blood cell lysing solution. The samples were resuspended in a staining buffer comprising 2% Hyclone in PBS, followed by cell count analysis. For surface staining, the samples were incubated with specific antibodies (see Table 1) in a staining buffer for 30 min at 4°C. For staining PBMCs, dead cells were marked with Fixable Viability Stain 780 (FVS780) and live cells were further analyzed. Cell staining was performed as follows: Total lymphocytes (CD45+), B cells (CD45+ CD45R + CD20+), total T cells (CD45+ CD3+), CD4+ T cells (CD45+ CD3+ CD4+), CD8+ T cells (CD45+ CD3+ CD8+), activated CD4+ T cells (CD45+ CD3+ CD4+ CD69+), activated CD8+ T cells (CD45+ CD3+ CD8+ CD69+), Treg cells (CD45+ CD3+ CD4+ CD25+), activated Treg cells (CD45+ CD3+ CD4+ CD25+ CD120b), NK cells (CD45+ CD3− NK1.1+), activated NK cells (CD45+ CD3− NK1.1+ CD69+). Flow cytometric analysis was performed using the FACSymphony A5 instrument (BD Biosciences, CA, USA). Data were analyzed using FlowJo software (Version 10.5.3, FlowJo LLC, OR, USA).

### 16S rRNA sequencing and bioinformatic analysis

After administering DcR3 for 12 h, mice were euthanized by cervical dislocation. Subsequently, their exterior was disinfected with a 75% alcohol solution, and their abdominal cavities were carefully opened to procure fecal samples from the cecum to the rectum segment. These fecal samples were meticulously transferred into sterile Eppendorf tubes, with each group consisting of five replicates for statistical robustness. Finally, the collected mouse fecal samples were dispatched to Beijing Biomarker Technologies Co., Ltd. for comprehensive analysis and determination. DNA extraction and 16S rRNA sequencing were performed as previously described (45). DNA was extracted using the Magnetic Soil and Stool DNA Kit (Cat. 4992738, Tiangen Biotech Co., Ltd.) per the manufacturer’s instructions. The full-length 16S rRNA gene was amplified from DNA using the universal primer set: 27F (5′-AGRGTTTGATYNTGGCTCAG-3′) and 1492R (5′-TASGGHTACCTTGTTASGACTT-3′), with sample-specific PacBio barcode sequences. PacBio Sequel II platform (Biomarker Ltd) was used for library construction, sequencing, and data analysis. The BMK Cloud (https://www.biocloud.net) was used for bioinformatics analysis, as previously described (45).

### Detection of SCFAs

SCFA extraction and detection were performed following a previously described method using gas chromatography-mass spectrometry (Shimadzu, Japan) (46).

### Whole-genome sequencing analysis

RNA sequencing (RNA-Seq) was conducted by Beijing BioMarker Bioinformatics Technology Co., Ltd. (Beijing, China), utilizing the Illumina NovaSeq 6000 platform. We adhered to the previously described protocol for comprehensive genome sequencing analysis (47).

### Statistical analysis

Images were processed using Photoshop, Illustrator 2020 (Adobe, San Jose, CA, USA), and ImageJ v1.8.0 (National Institutes of Health, Bethesda, MD, USA). Statistical analyses were performed using GraphPad Prism (v8.0; GraphPad Software, San Diego, CA, USA). The unpaired two-tailed *t*-test, one-way analysis of variance (ANOVA), two-way ANOVA, or Mantel-Cox test were used for statistical analysis. Data are expressed as mean ± standard deviation. Differences were considered significant at *P* < 0.05.

## Data Availability

The data referred to in this study can be accessed at https://ngdc.cncb.ac.cn/gsa/s/Sv08k116 and https://ngdc.cncb.ac.cn/gsa/s/WcfI5sKa.

## SUPPLEMENTAL MATERIAL

Supplemental Material mbio.00521-24-s0001.docx Supplemental text and figures.

ASM does not own the copyrights to Supplemental Material that may be linked to, or accessed through, an article. The authors have granted ASM a non-exclusive, world-wide license to publish the Supplemental Material files. Please contact the corresponding author directly for reuse.
