# Supplementary material for: Inhibitory mechanisms of decoy receptor 3 in cecal ligation and puncture-induced sepsis
Source: mBio. 2024 May 3;15(6):e00521-24. doi: 10.1128/mbio.00521-24 (PMC11237498; doi:10.1128/mbio.00521-24)
Supplement: Supplemental Material — Supplemental text and figures. [file mbio.00521-24-s0001.docx]

**Supplemental Material For**

**Inhibitory Mechanisms of Decoy Receptor 3 in Cecal Ligation and Puncture-induced Sepsis**

**Supplemental Figures**

GGATCCGTTGCCGAAACCCCGACCTATCCGTGGCGCGATGCCGAAACCGGCGAACGTCTGGTGTGCGCCCAGTGCCCGCCGGGTACATTTGTTCAGCGTCCGTGCCGTCGTGATAGTCCGACCACCTGCGGTCCGTGCCCGCCTAGACATTATACCCAGTTTTGGAATTATCTGGAACGCTGTCGTTATTGTAATGTTCTGTGCGGCGAACGTGAAGAAGAAGCCCGTGCCTGTCATGCAACCCATAATCGCGCCTGCCGTTGCCGTACCGGCTTTTTCGCACATGCCGGTTTTTGCCTGGAACATGCCAGCTGTCCGCCGGGTGCCGGTGTGATTGCCCCTGGTACACCGAGCCAGAATACCCAGTGTCAGCCGTGCCCGCCGGGCACATTTTCAGCCAGCAGCAGCAGCAGTGAACAGTGTCAGCCTCATCGCAATTGTACCGCCCTGGGTCTGGCCCTGAATGTTCCGGGTAGTAGCAGCCATGATACCCTGTGTACCAGCTGTACCGGCTTTCCGCTGAGCACCCGCGTGCCGGGTGCCGAGGAATGTGAACGTGCCGTTATTGATTTTGTGGCCTTTCAGGATATTAGCATTAAGCGCCTGCAACGTCTGCTGCAAGCCCTGGAAGCACCGGAAGGTTGGGGCCCGACCCCGAGAGCTGGCAGAGCTGCTCTGCAACTGAAACTGCGCCGTCGTCTGACCGAACTGCTGGGTGCACAGGATGGCGCCCTGCTGGTTCGTCTGCTGCAAGCGCTGCGTGTTGCACGTATGCCGGGTCTGGAACGTAGTGTTCGTGAACGTTTTCTGCCGGTTCATTAACTCGAG

**Fig. S1. The sequence alignment of DcR3.**


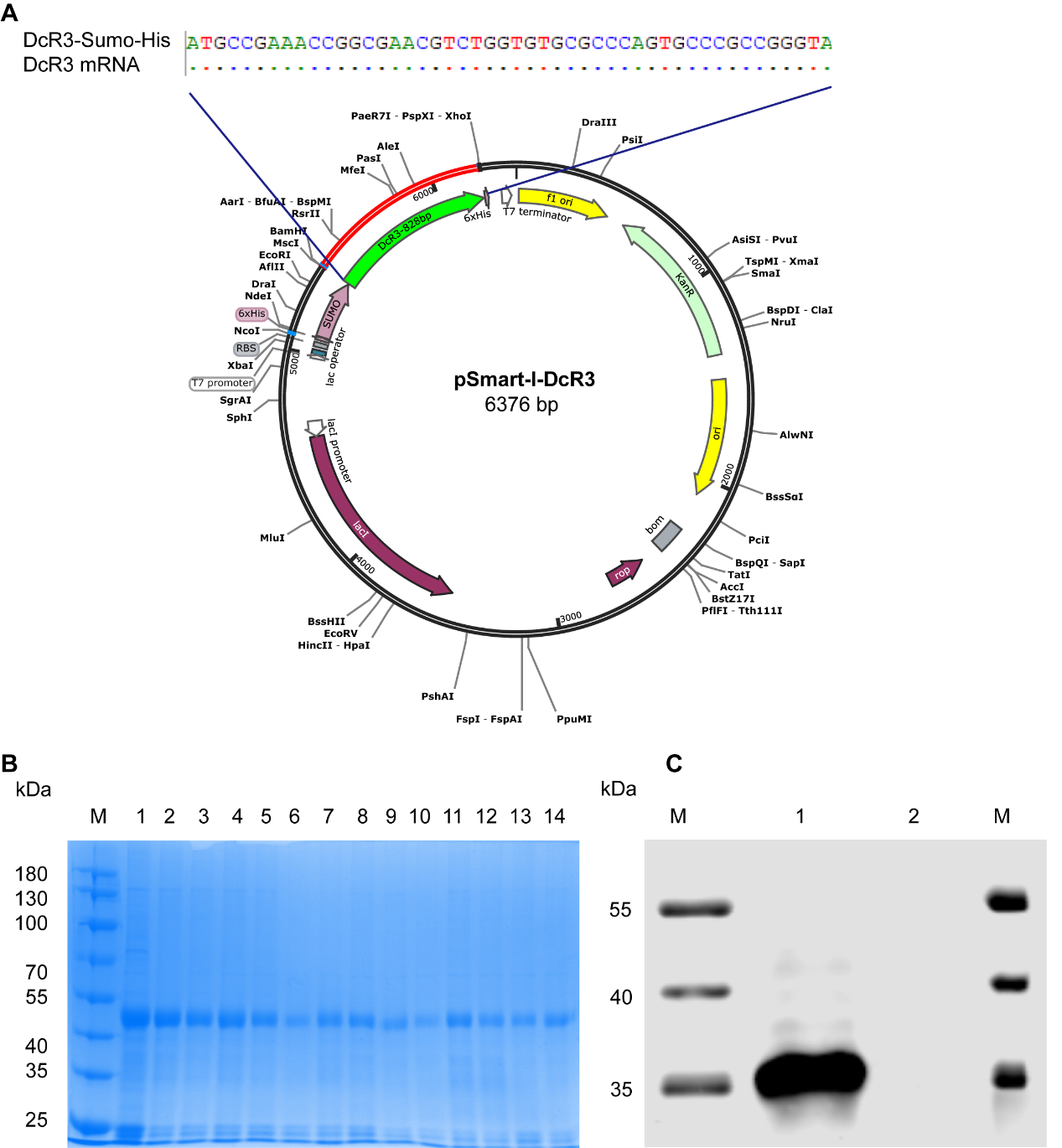


**Fig.S2. Purification and identification of DcR3.** (A) Schematic representation of the cloned DcR3 in the pSmart-I vector. (B) Bacterially purified His-DcR3 using SDS-PAGE. (C) Western blot assay with aHis; lane 1 is the unmodified Sumo-His tag DcR3 protein, and lane 2 is the Sumo-His tag excised DcR3 protein.


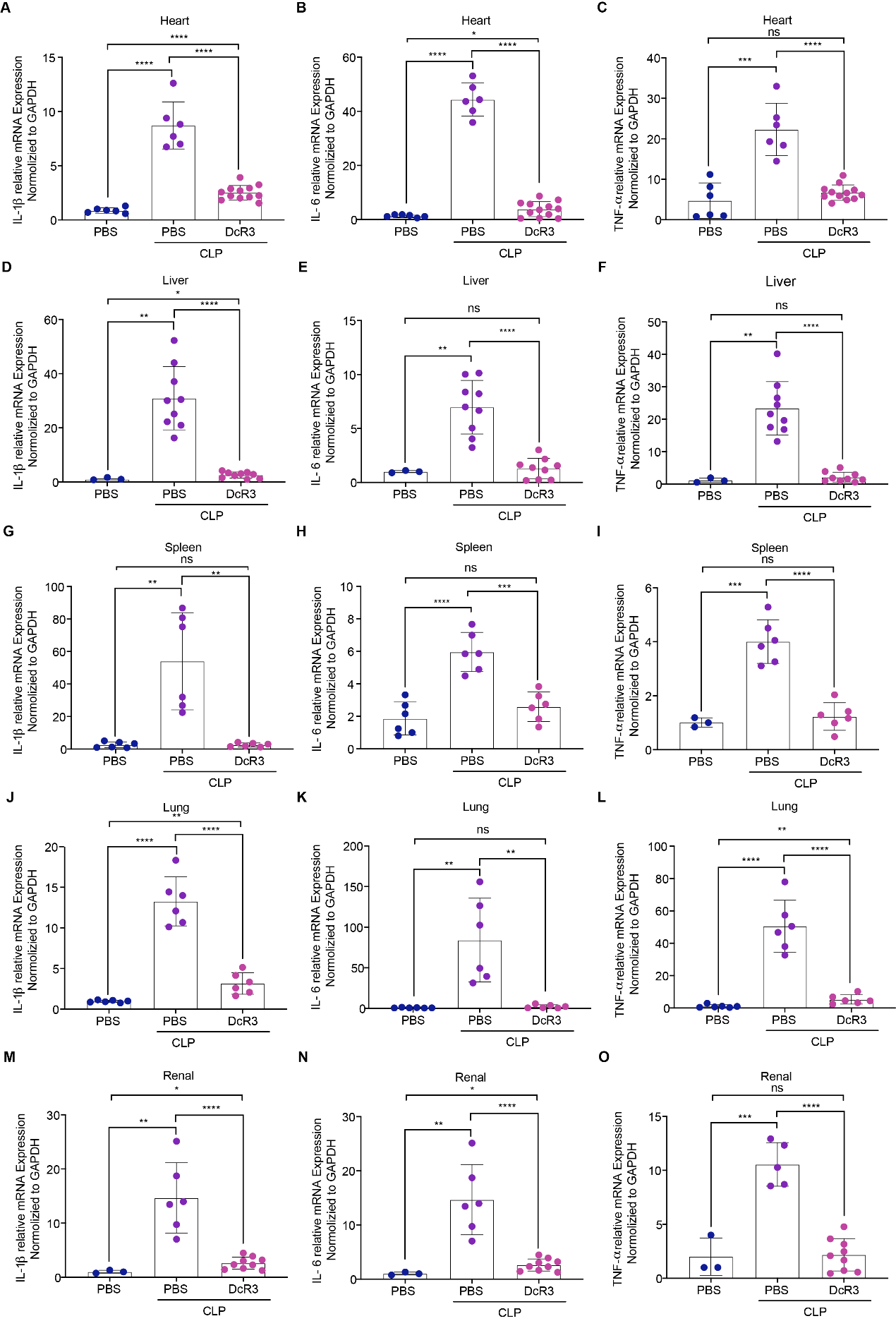


**Fig. S3**. **A 12 h DcR3 treatment reduced proinflammatory cytokine levels in CLP-induced sepsis mice.** The mRNA expression of IL-1β, IL-6, and TNF-α in the (A-C) heart, (D-F) liver, (G-I) spleen, (J-L) lung, and (M-O) kidney tissues of septic mice were measured using qRT-PCR after 12 h of DcR3 treatment. ANOVA and Tukey’s post hoc test were performed to analyze the data. (∗) *P* < 0.05, (∗∗) *P* < 0.01, (∗∗∗) *P* < 0.001, and (∗∗∗∗) *P* < 0.0001; ns, not significant (*P* > 0.05). An independent experiment was conducted three times to produce the results.

**
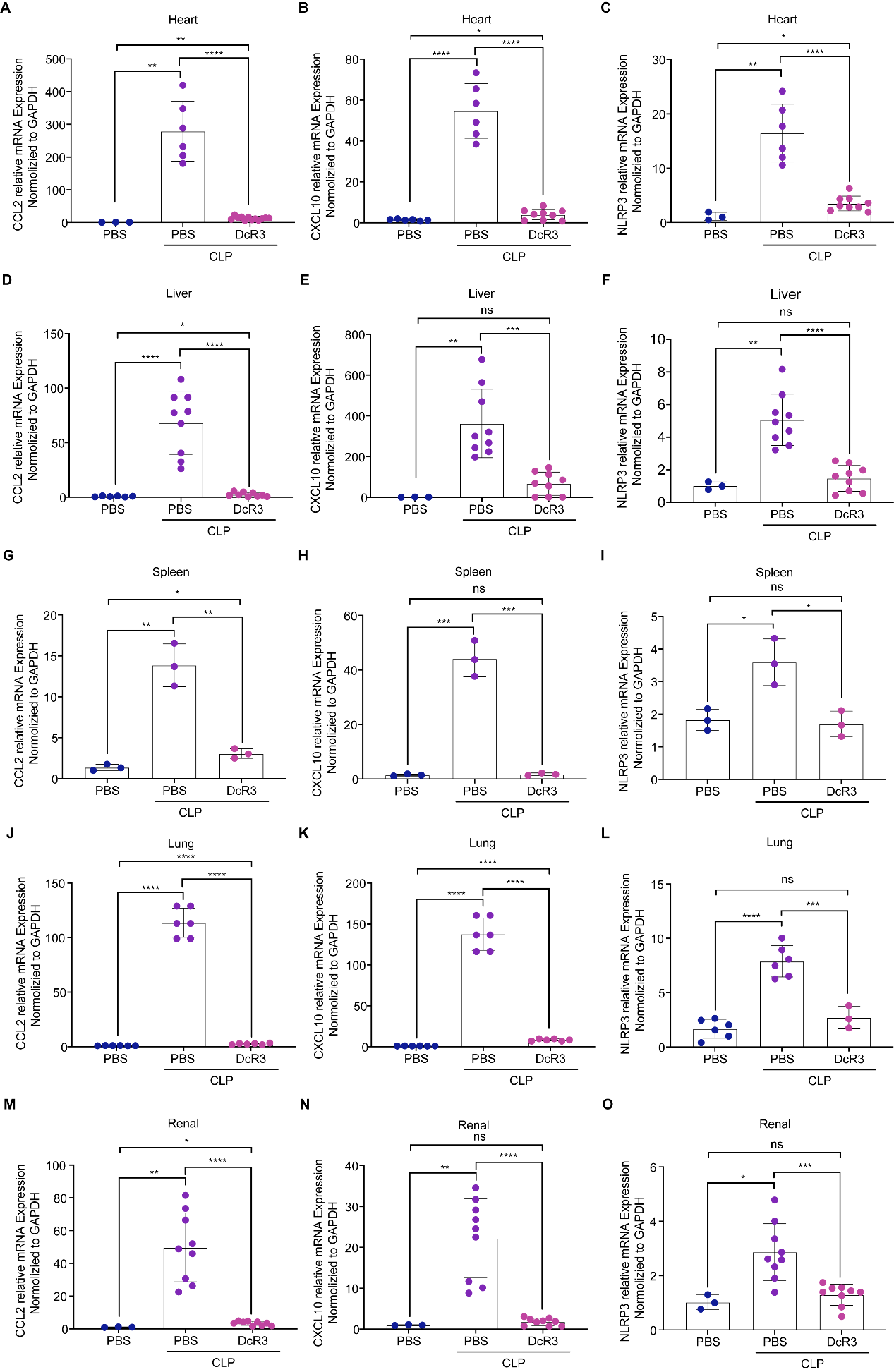
Fig. S4. A12 h DcR3 treatment reduced proinflammatory factor levels in septic mice.** The mRNA expression of CCL2, CXCL10, and NLRP3 in the (A-C) heart, (D-F) liver, (G-I) spleen, (J-L) lung, and (M-O) kidney tissues of mice with sepsis were measured using qRT-PCR at 24 h after DcR3 treatment. ANOVA and Tukey's post hoc test were performed to analyze the data. (∗) *P* < 0.05, (∗∗) *P* < 0.01, (∗∗∗) *P* < 0.001, and (∗∗∗∗) *P* < 0.0001; ns, not significant (*P* > 0.05). An independent experiment was conducted three times to produce the results.


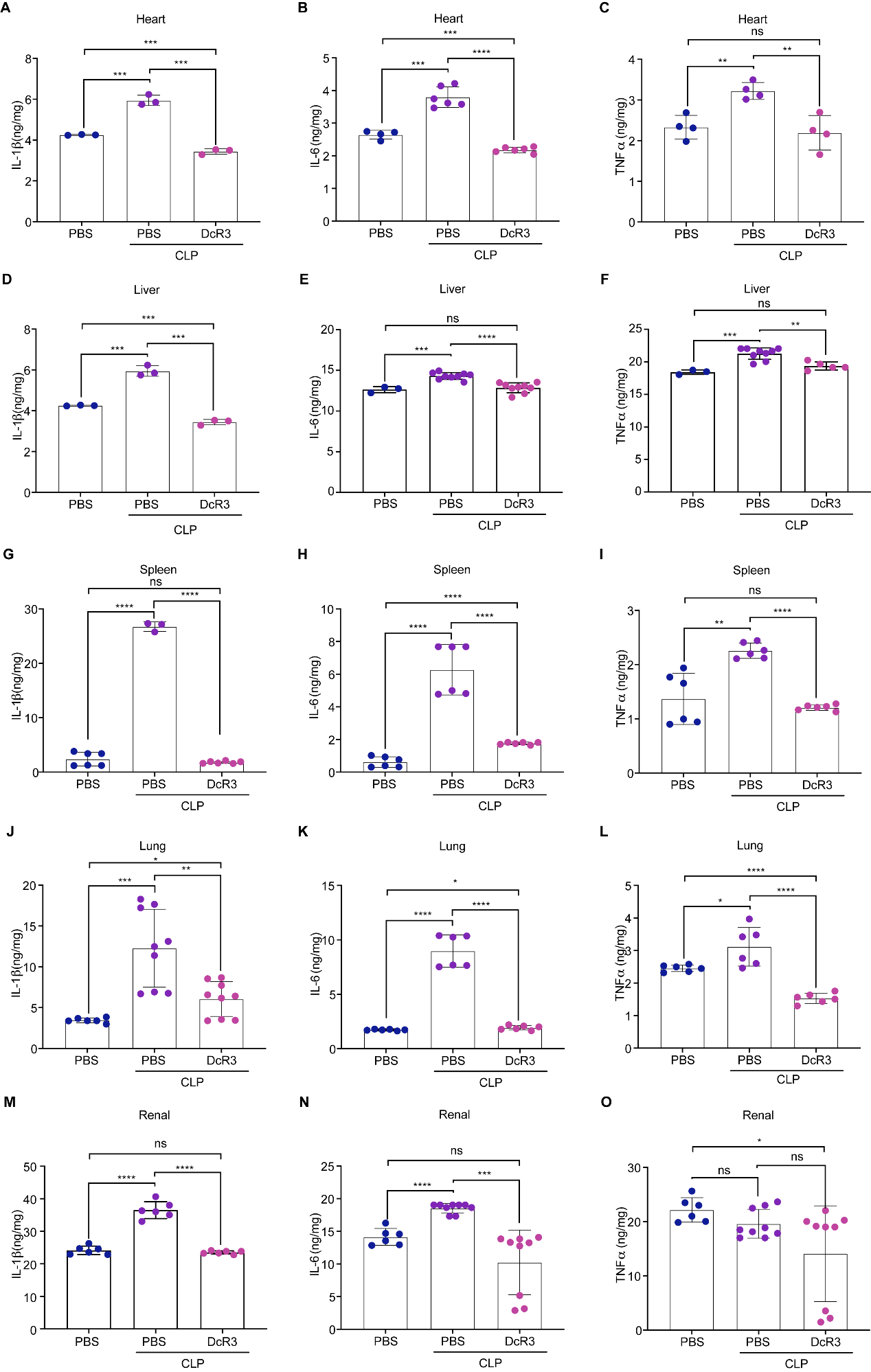


**Fig. S5**. **Changes in inflammatory cytokines determined using ELISA** **in CLP-induced sepsis mice at 12 h after the DcR3 treatment.** The levels of IL-1β, IL-6, and TNF-α in the (A-C) heart, (D-F) liver, (G-I) spleen, (J-L) lung, and (M-O) kidney tissues of septic mice were measured using ELISA at 12 h after the DcR3 treatment. ANOVA and Tukey's post hoc test were performed to analyze the data. (∗) *P* < 0.05, (∗∗) *P* < 0.01, (∗∗∗) *P* < 0.001, and (∗∗∗∗) *P* < 0.0001; ns, not significant (*P* > 0.05). An independent experiment was conducted three times to produce the results.


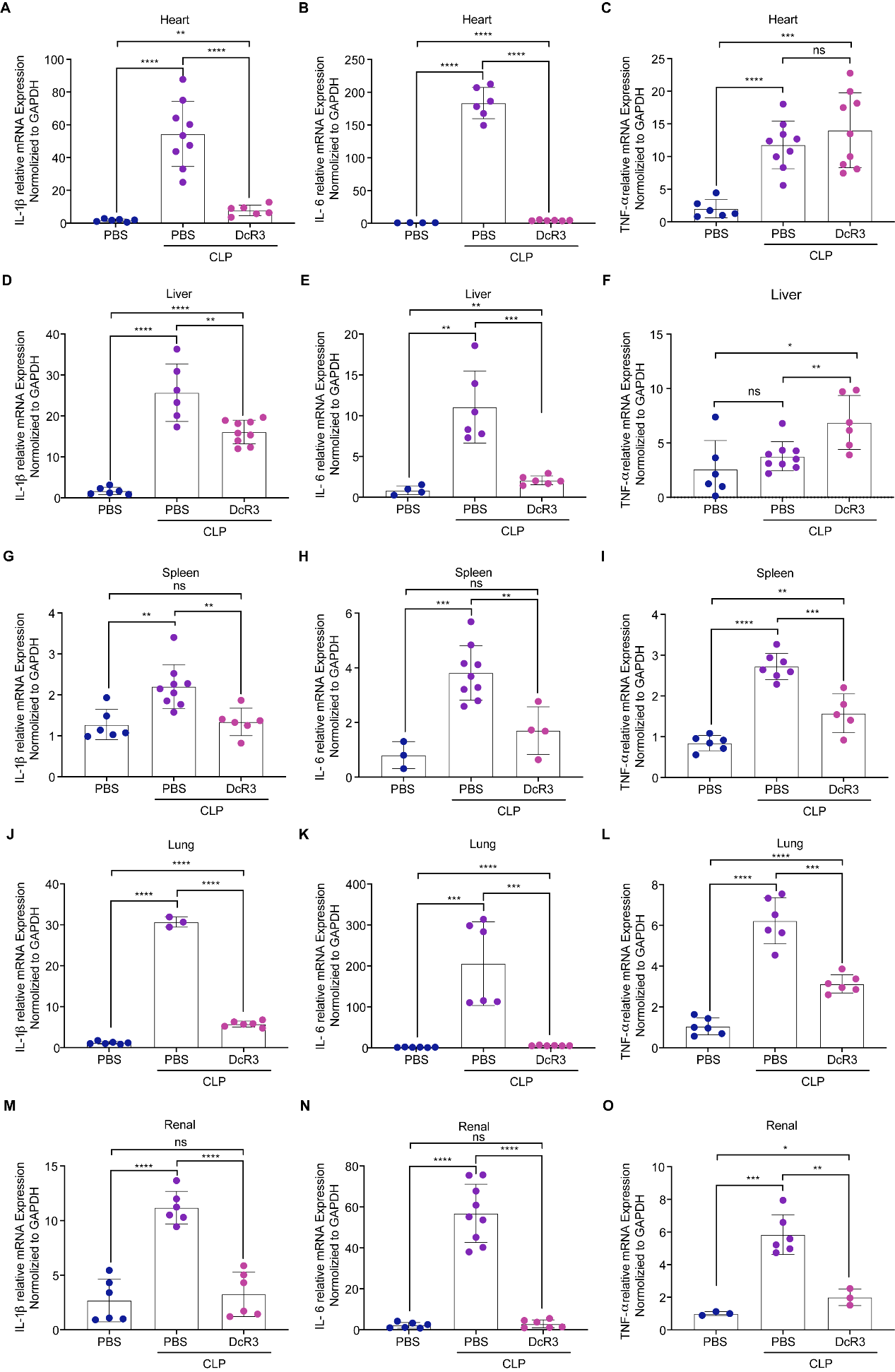
**Fig. S6**. **A 24 h DcR3 treatment reduced proinflammatory cytokine levels in septic mice.** The mRNA expression of IL-1β, IL-6, and TNF-α in the (A-C) heart, (D-F) liver, (G-I) spleen, (J-L) lung, and (M-O) kidney tissues of septic mice were measured using qRT-PCR at 24 h after the DcR3 treatment. ANOVA and Tukey’s post hoc test were performed to analyze the data. (∗) *P* < 0.05, (∗∗) *P* < 0.01, (∗∗∗) *P* < 0.001, and (∗∗∗∗) *P* < 0.0001; ns, not significant (*P* > 0.05). An independent experiment was conducted three times to produce the results.


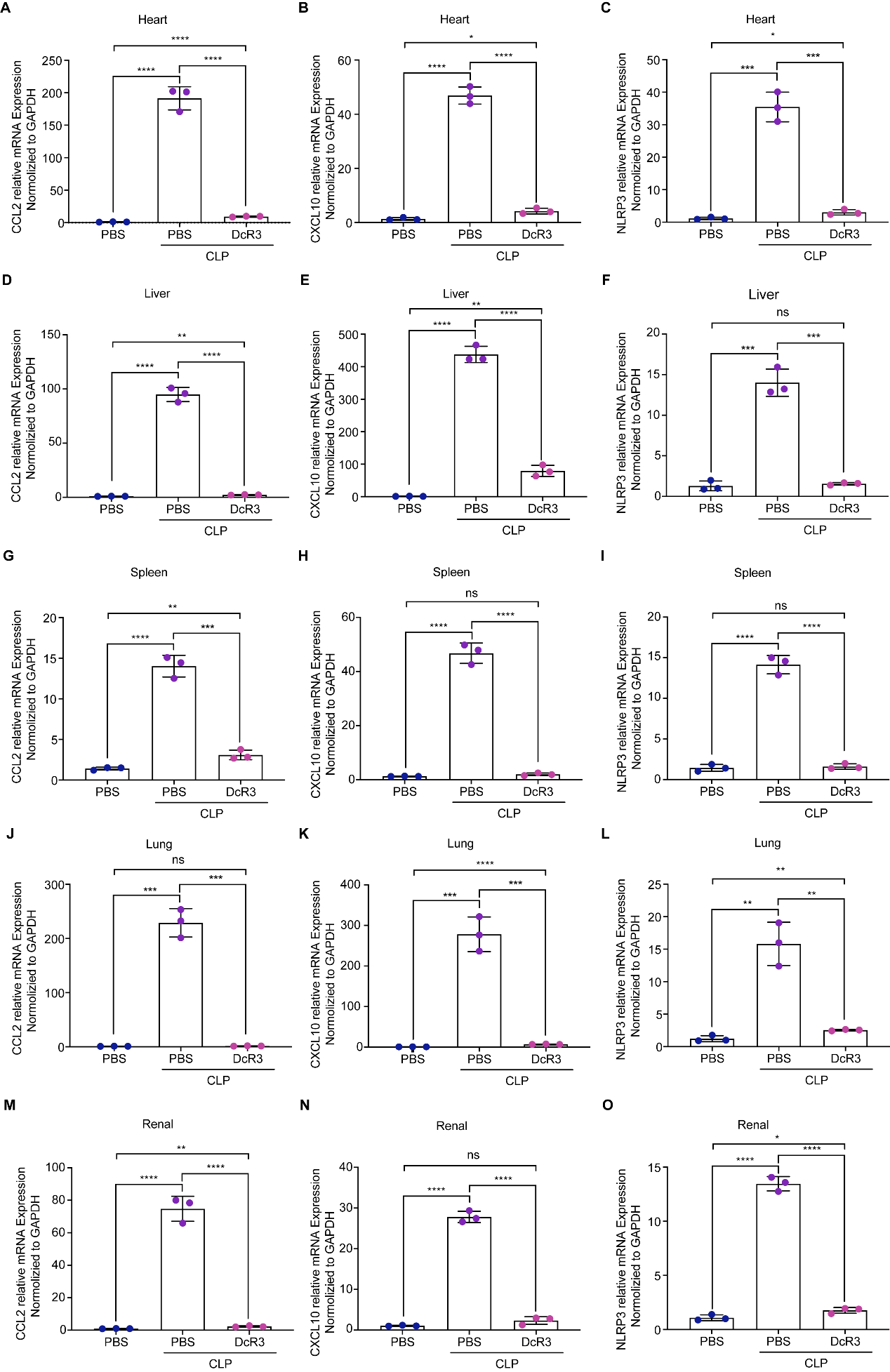
**Fig. S7. A 24 h DcR3 treatment reduced proinflammatory factor levels in septic mice.** The mRNA expression of CCL2, CXCL10, and NLRP3 in the (A-C) heart, (D-F) liver, (G-I) spleen, (J-L) lung, and (M-O) kidney tissues of mice with sepsis were measured using qRT-PCR at 24 h after DcR3 treatment. ANOVA and Tukey's post hoc test were performed to analyze the data. (∗) *P* < 0.05, (∗∗) *P* < 0.01, (∗∗∗) *P* < 0.001, and (∗∗∗∗) *P* < 0.0001; ns, not significant (*P* > 0.05). An independent experiment was conducted three times to produce the results.


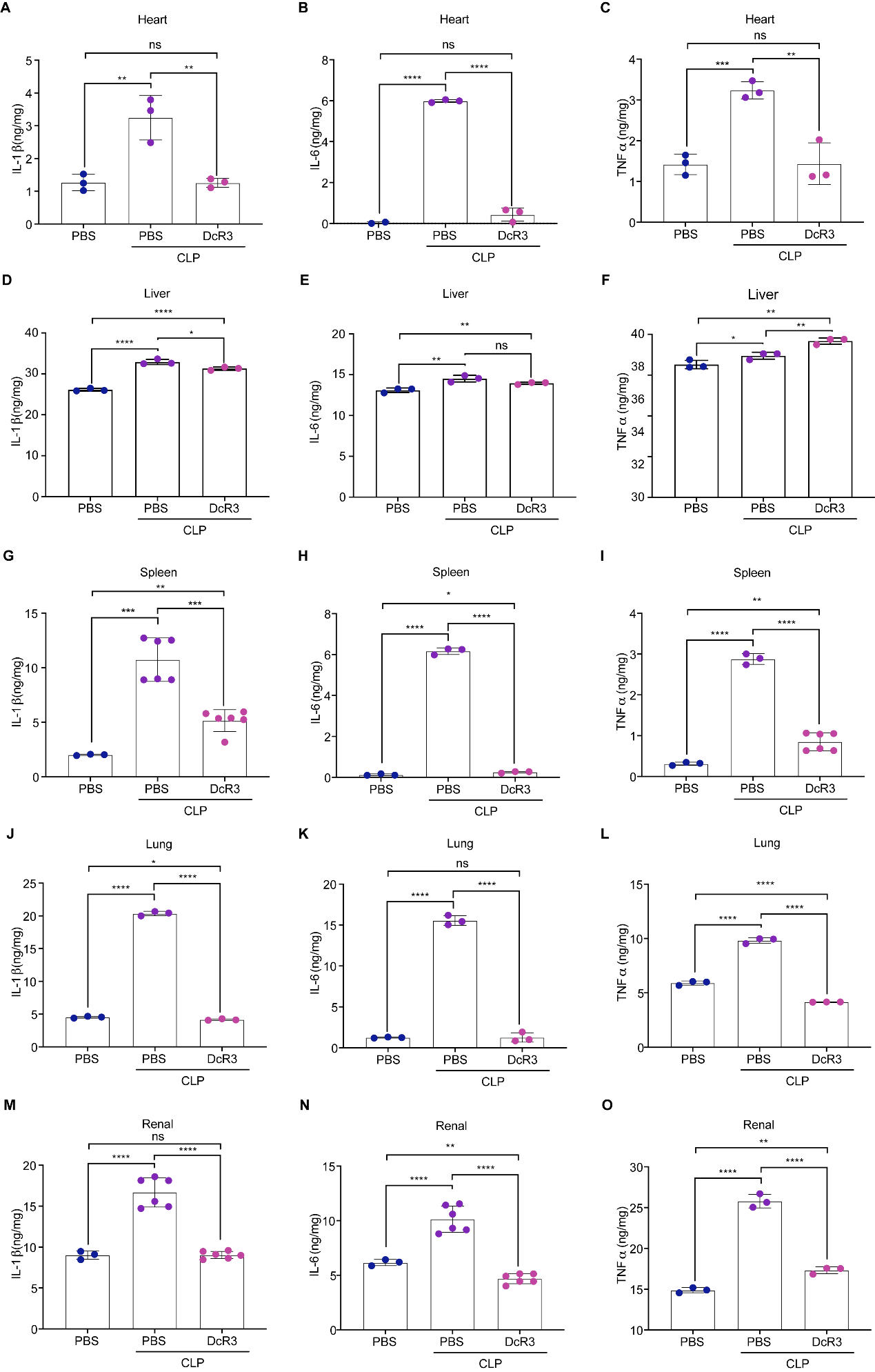


**Fig. S8**. **Changes in inflammatory cytokines determined using ELISA** **in mice with sepsis at 24 h after the DcR3 treatment.** The levels of IL-1β, IL-6, and TNF-α in the (A-C) heart, (D-F) liver, (G-I) spleen, (J-L) lung, and (M-O) kidney tissues of septic mice were measured using ELISA at 24 h after the DcR3 treatment. ANOVA and Tukey's post hoc test were performed to analyze the data. (∗) *P* < 0.05, (∗∗) *P* < 0.01, (∗∗∗) *P* < 0.001, and (∗∗∗∗) *P* < 0.0001; ns, not significant (*P* > 0.05). An independent experiment was conducted three times to produce the results.


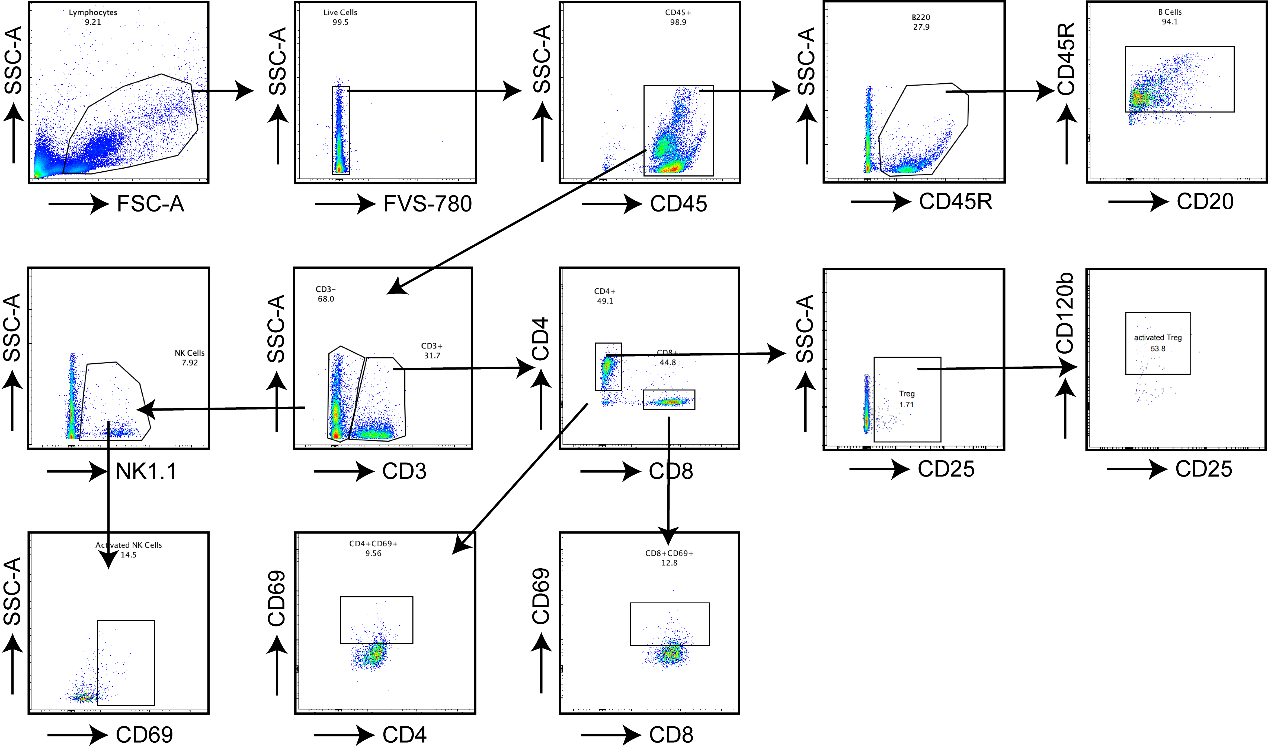


**Fig. S9. Gating strategies for flow cytometry.**


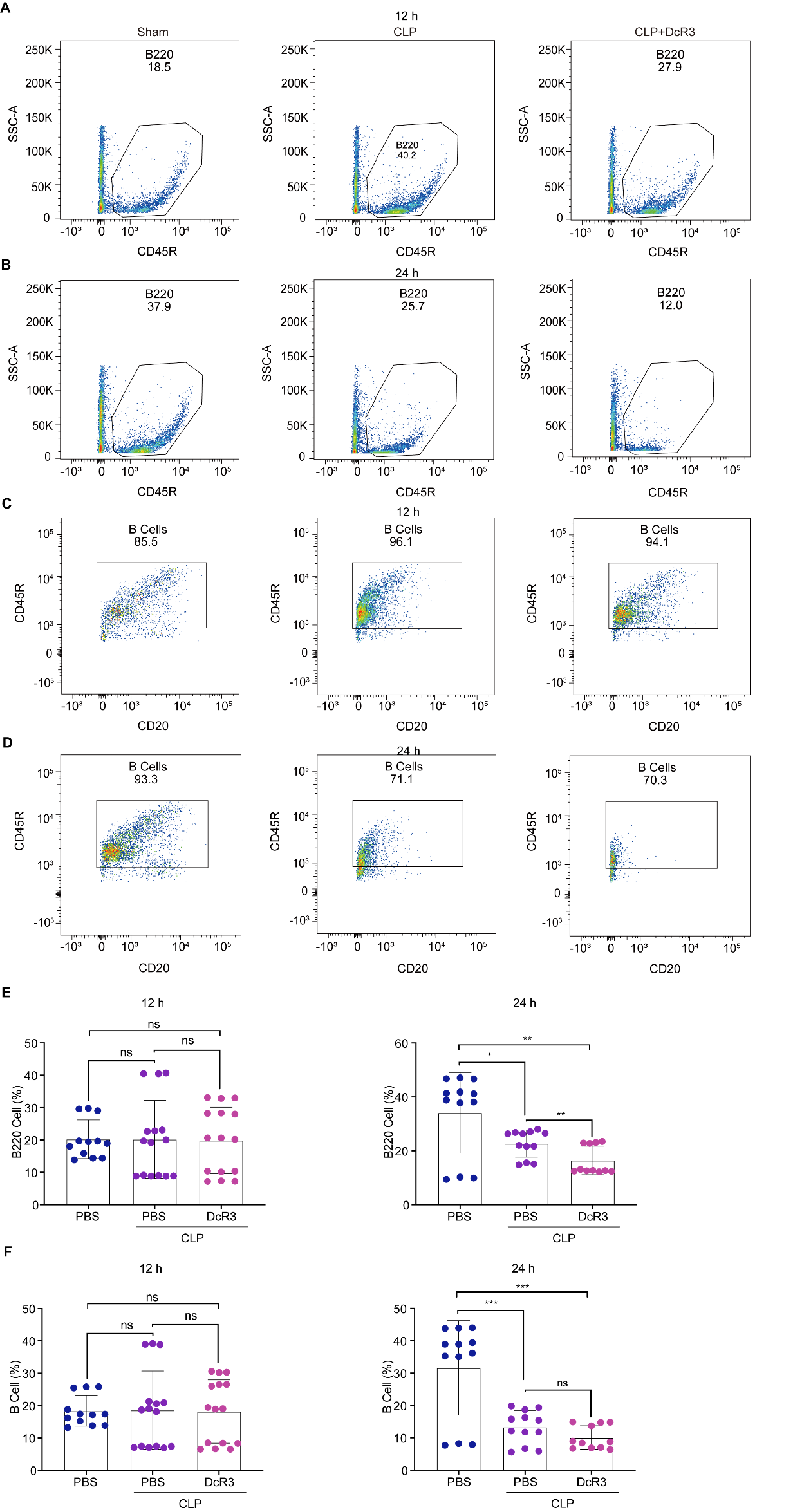


**Fig. S10. Effects of DcR3 12 and 24 h treatments on the expression of B220 and B cells in peripheral blood of CLP-induced sepsis mice.** (A-D) Flow chart and detection markers for B220 and B cells assessed using flow cytometry in CLP-induced sepsis mice at 12 and 24 h after the DcR3 treatment. (E, F) Number of B220 cells at 12 and 24 h. (G, H) Number of B cells at 12 and 24 h. ANOVA and Tukey’s post hoc test were performed to analyze the data. (∗) *P* < 0.05, (∗∗) *P* < 0.01, (∗∗∗) *P* < 0.001, and (∗∗∗∗) *P* < 0.0001; ns, not significant (*P* > 0.05). An independent experiment was conducted three times to produce the results.


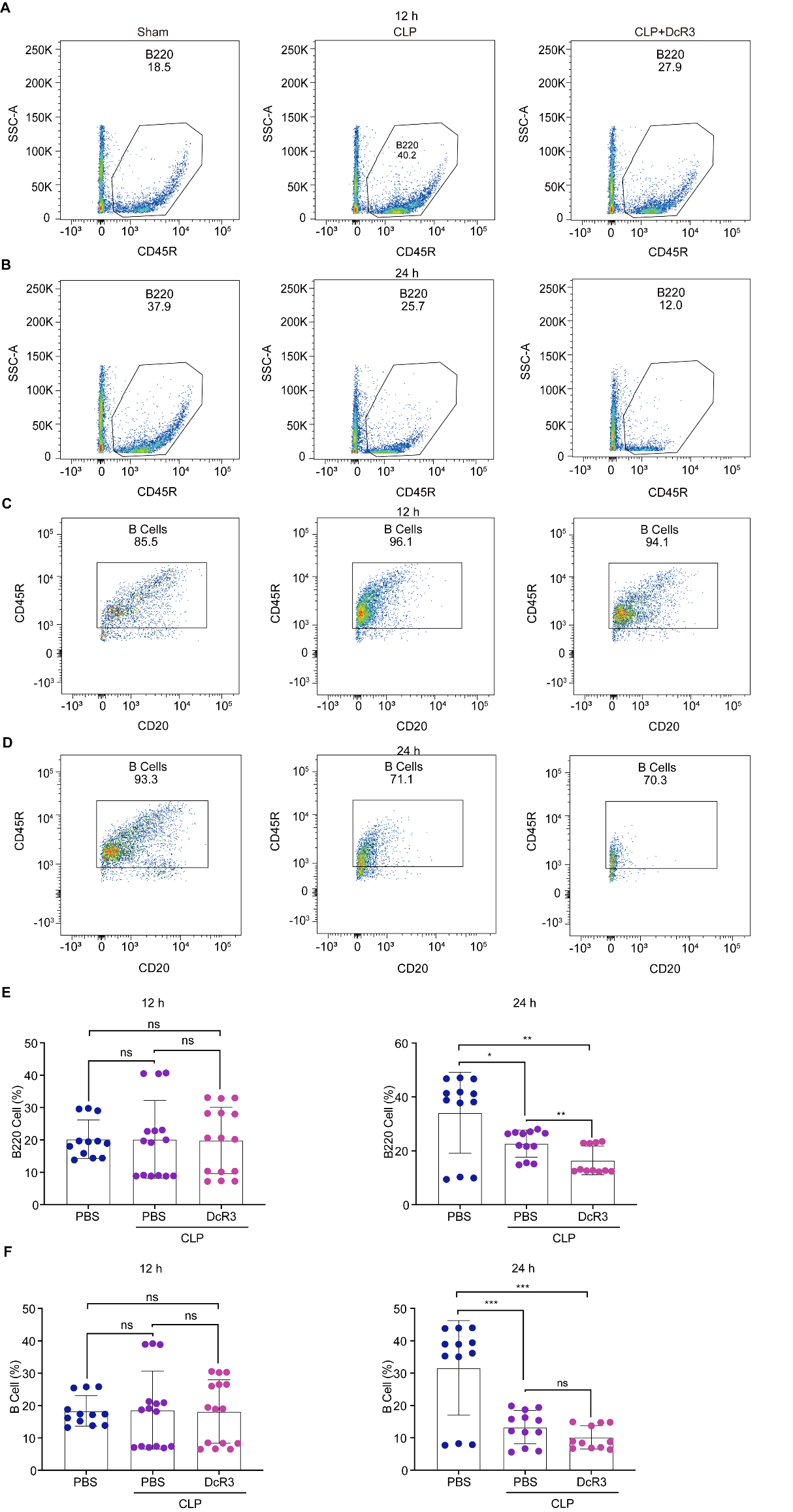


**Fig. S11. Effects of DcR3 12 and 24 h treatments on the expression of CD4^+^ T and CD8^+^ T cells in peripheral blood of CLP-induced sepsis mice.** (A, B) Flow chart and detection markers for CD4^+^ T and CD8^+^ T cells were assessed using flow cytometry in CLP-induced sepsis mice at 12 and 24 h. (C) Number of CD4^+^ T cells at 12 and 24 h. (D) Number of CD8^+^ T cells at 12 and 24 h. ANOVA and Tukey's post hoc test were performed to analyze the data. (∗) *P* < 0.05, (∗∗) *P* < 0.01, (∗∗∗) *P* < 0.001, and (∗∗∗∗) *P* < 0.0001; ns, not significant (*P* > 0.05). An independent experiment was conducted three times to produce the results.


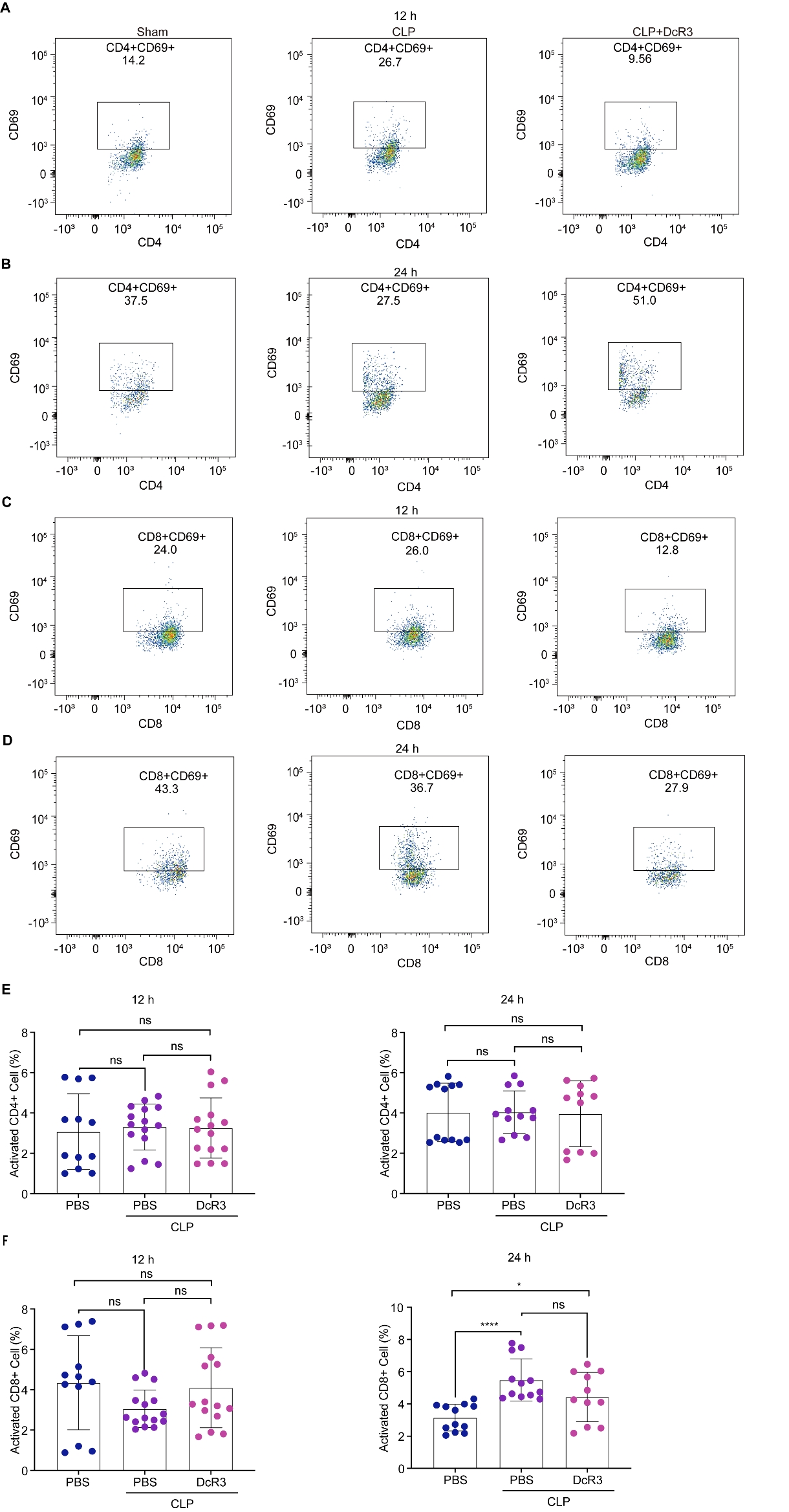


**Fig. S12. Effects of DcR3 12 and 24 h treatments on the expression of activated CD4^+^ T and activated CD8^+^ T cells in peripheral blood of CLP-induced sepsis mice.** (A, B) Flow chart and detection markers for activated CD4^+^ T cells were assessed using flow cytometry in CLP-induced sepsis mice at 12 and 24 h. (C, D) Flow chart and detection markers for activated CD8^+^ T cells were assessed using flow cytometry in CLP-induced sepsis mice at 12 and 24 h. (E) Number of activated CD4^+^ T cells at 12 and 24 h. (F) Number of activated CD8^+^ T cells at 12 and 24 h. ANOVA and Tukey's post hoc test were performed to analyze the data. (∗) *P* < 0.05; ns, not significant (*P* > 0.05). An independent experiment was conducted three times to produce the results.


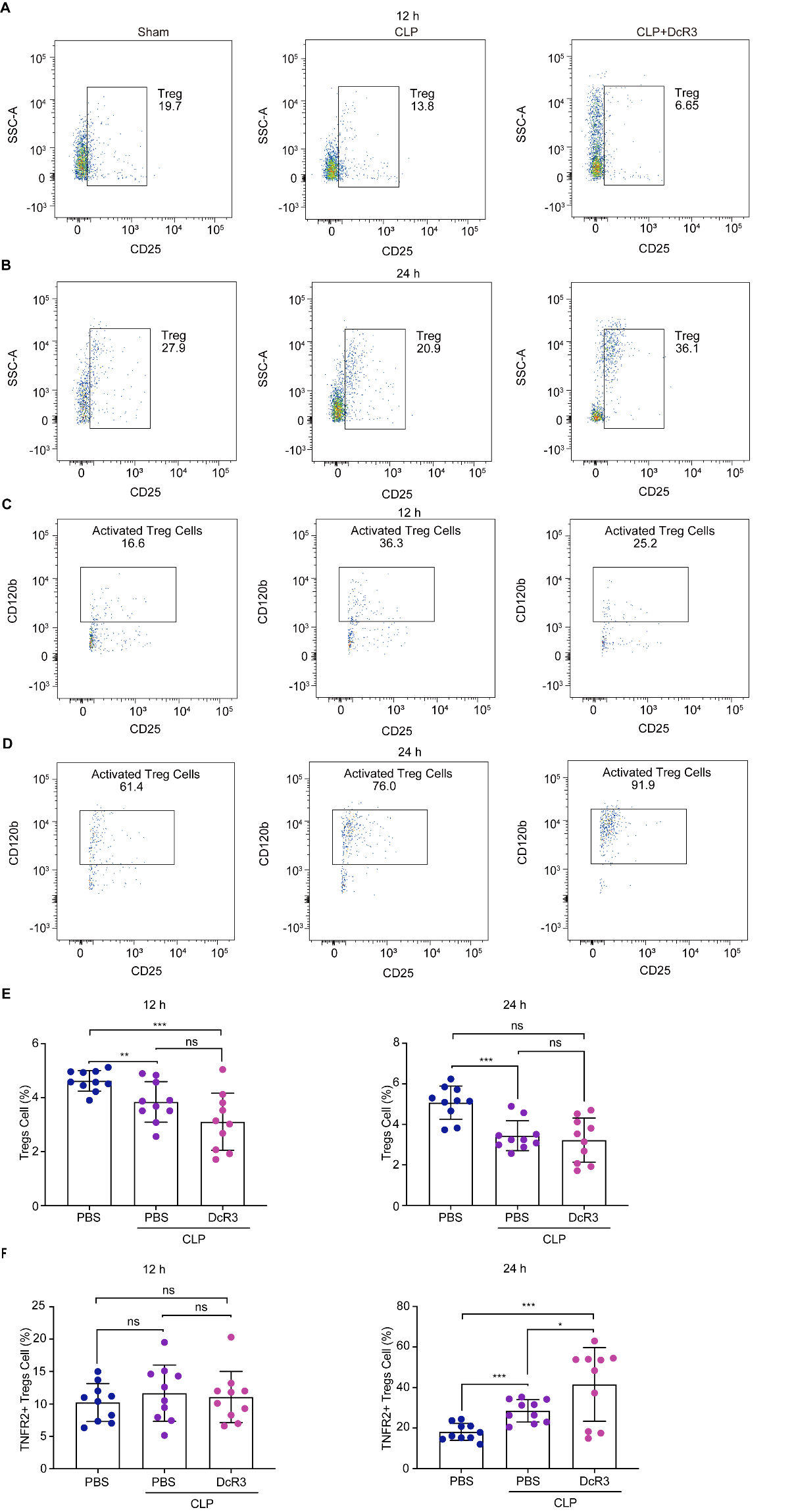


**Fig. S13. Effects of DcR3 12 and 24 h treatments on the expression of Treg and activated Treg cells in peripheral blood of CLP-induced sepsis mice.** (A-D) Flow chart and detection markers for Treg and activated Treg cells were assessed using flow cytometry in CLP-induced sepsis mice at 12 and 24 h. (E) Number of Treg cells at 12 and 24 h. (F) Number of activated Treg at 12 and 24 h. ANOVA and Tukey's post hoc test were performed to analyze the data. (#) *P* < 0.05 and (##) *P* < 0.01 vs. the control group; ns, not significant. (∗) *P* < 0.05, (∗∗) *P* < 0.01, (∗∗∗) *P* < 0.001, and (∗∗∗∗) *P* < 0.0001; ns, not significant (*P* > 0.05). An independent experiment was conducted three times to produce the results.


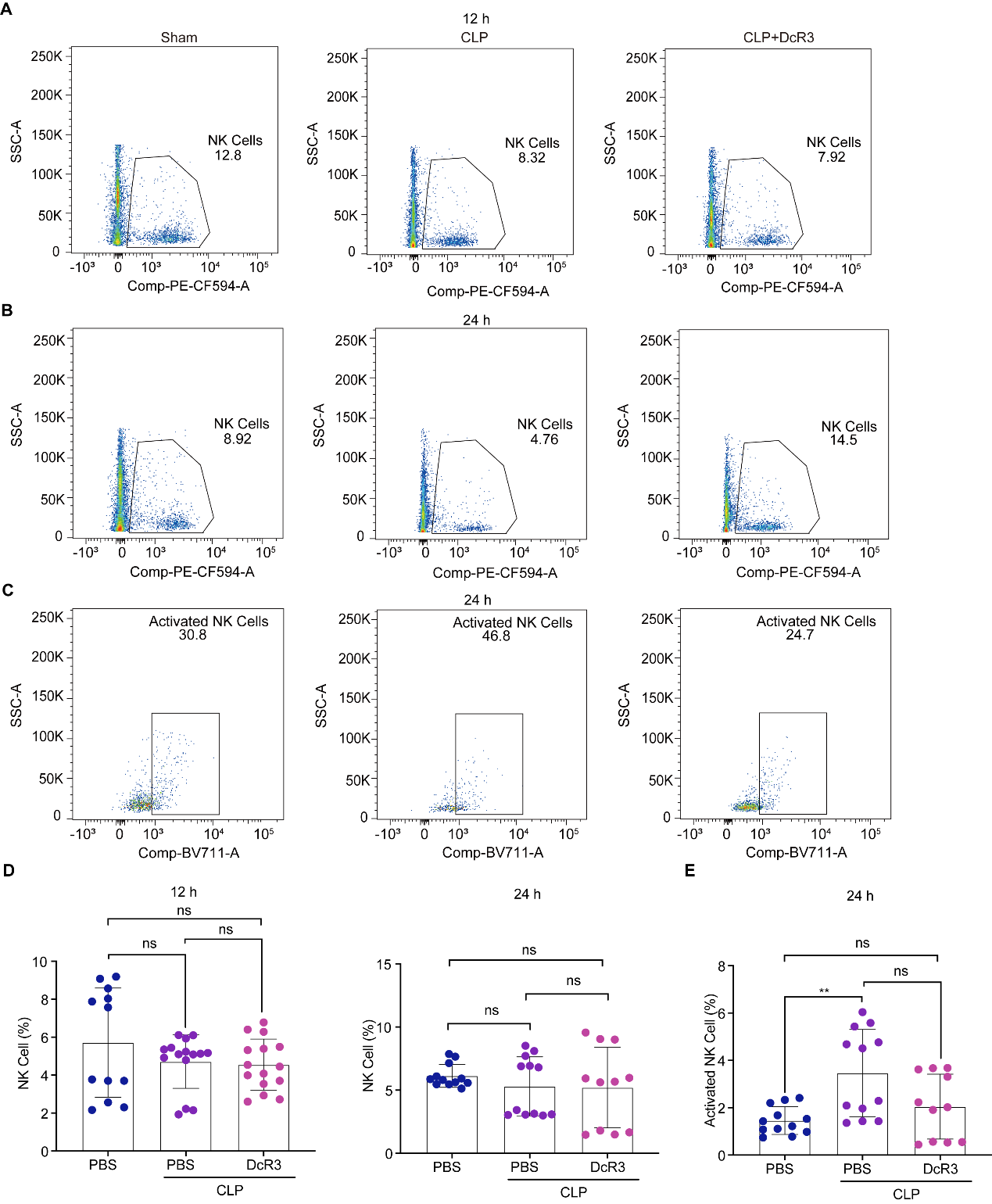


**Fig. S14. Effects of DcR3 12 and 24 h treatments on the expression of NK and activated NK cells in peripheral blood of CLP-induced sepsis mice.** (A-C) Flow chart and detection markers for NK and activated NK cells were assessed using flow cytometry in CLP-induced sepsis mice at 12 and 24 h. (D) Number of NK cells at 12 and 24 h. € Number of activated NK at 24 h. ANOVA and Tukey's post hoc test were performed to analyze the data. (∗) *P* < 0.05, (∗∗) *P* < 0.01, (∗∗∗) *P* < 0.001, and (∗∗∗∗) *P* < 0.0001; ns, not significant (*P* > 0.05). An independent experiment was conducted three times to produce the results.


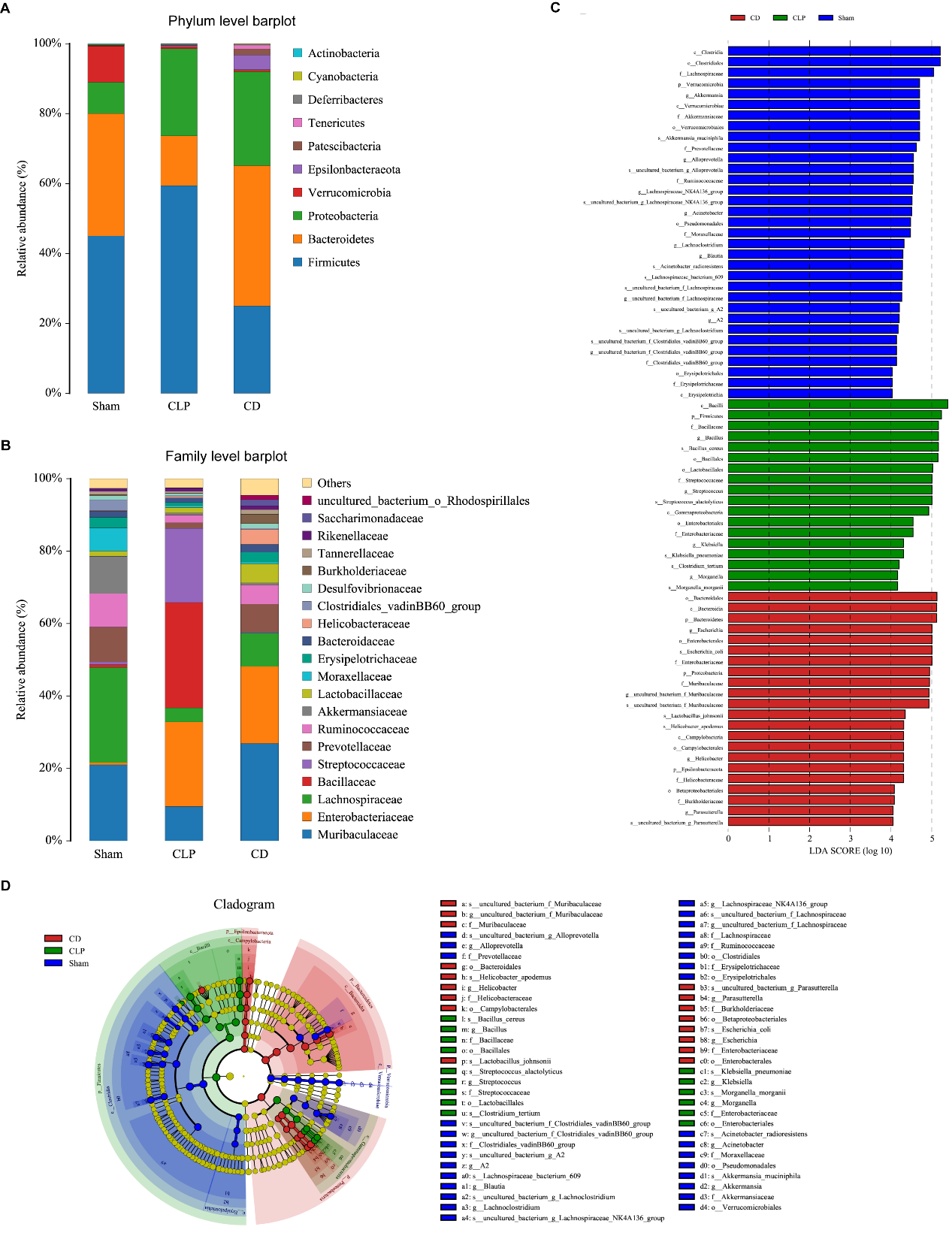


**Fig. S15. DcR3 treatment alleviates gut microbiota dysbiosis in CLP-induced sepsis mice.** Composition of gut microbiota at the (A) phylum and (B) family levels. (C) LEfSe comparison of the gut microbiota (LDA > 4.0); (D) Cladogram generated based on the LEfSe analysis.
